# MicroRNA regulation in colorectal cancer tissue and serum

**DOI:** 10.1371/journal.pone.0222013

**Published:** 2019-08-30

**Authors:** Lukasz Gmerek, Kari Martyniak, Karolina Horbacka, Piotr Krokowicz, Wojciech Scierski, Pawel Golusinski, Wojciech Golusinski, Augusto Schneider, Michal M. Masternak

**Affiliations:** 1 College of Medicine, Burnett School of Biomedical Sciences, University of Central Florida, Orlando, FL, United States of America; 2 Department of General and Colorectal Surgery, Poznan University of Medical Sciences, Poznan, Poland; 3 Department of Otorhinolaryngology and Laryngological Oncology in Zabrze, Medical University of Silesia, Katowice, Poland; 4 Department of Otolaryngology and Maxillofacial Surgery, University of Zielona Gora, Zielona Gora, Poland; 5 Department of Head and Neck Surgery, Poznan University of Medical Sciences, The Greater Poland Cancer Centre, Poznan, Poland; 6 Faculdade de Nutrição, Universidade Federal de Pelotas, Pelotas, RS, Brazil; Universitat des Saarlandes, GERMANY

## Abstract

Colorectal cancer is recognized as the fourth leading cause of cancer-related deaths worldwide. Thus, there is ongoing search for potential new biomarkers allowing quicker and less invasive detection of the disease and prediction of the treatment outcome. Therefore, the aim of our study was to identify colorectal cancer specific miRNAs expressed in cancerous and healthy tissue from the same patient and to further correlate the presence of the same miRNAs in the circulation as potential biomarkers for diagnosis. In the current study we detected a set of 40 miRNAs differentially regulated in tumor tissue when comparing with healthy tissue. Additionally, we found 8 miRNAs differentially regulated in serum of colorectal cancer patients. Interestingly, there was no overlap in miRNAs regulated in tissue and serum, suggesting that serum regulated miRNAs may be not actively secreted from colorectal tumor cells. However, four of differentially expressed miRNAs, including miR-21, miR-17, miR-20a and miR-32 represent the miRNAs characteristic for different tumor types, including breast, colon, lung, pancreas, prostate and stomach cancer. This finding suggests important groups of miRNAs which can be further validated as markers for diagnosis of tumor tissue and regulation of carcinogenesis.

## Introduction

Cancer development encompasses alterations in cell growth, differentiation and regulation of apoptosis. Over a decades of cancer research many oncogenes and tumor suppressor genes have been identified and extensively studied for its role in the pathogenesis and malignancy of different types of cancer [1, 2]. In this scenario, the discovery of short small non-coding RNAs (sncRNAs) unveiled new potential molecular regulators of tumorigenesis [3]. MicroRNAs (miRNAs) are a class of sncRNAs that interact with the RNA Induced Silencing Complex (RISC) to bind to the 3’ untranslated region (UTR) of mRNA molecules and regulate transcription and mRNA stability [4, 5]. miRNAs have been shown to have an active role in cell growth and proliferation, being also implicated in tumorigenesis by regulating oncogenes and tumor suppressor genes expression [6, 7]. Tumor produced miRNAs are also regarded as predictors of malignancy and response to chemotherapy [3, 6].

miRNAs are produced in the nucleus and regulate gene expression in the cytoplasm of the cell [4]. However, miRNAs can be also found in the extracellular environment, including in serum, suggesting that it does not have an exclusively intracellular role [8–10]. The origin of extracellular miRNAs may include passive leakage from apoptotic or damaged cells and/or through secretory activity mainly within extracellular vesicles which includes exosomes [11]. Circulating miRNAs can have a role in intercellular communication, affecting gene expression in distant or adjacent target cells [11], or serve as biomarkers for pathological conditions [8]. Therefore, it is hypothesized that the signature of circulating miRNAs provide high sensitivity, success and reproducibility in the diagnostics of different types of cancer using a non-invasive approaches [8, 12, 13]. Despite previous work on miRNA signatures in serum or tissue of various types of cancer, including colorectal, very few studies approach tissue and serum variations of miRNAs simultaneously in the same patients. This paired method can suggest if the changes in circulating miRNA signatures are derived from the main tumoral tissue or are due secondary causes.

Due to high rate of colorectal cancer-related deaths worldwide [14, 15], the miRNA profile in biopsies and serum has been extensively studied for this condition [16–20] but the lack of more comprehensive studies in both tissue and serum from the same patients and the repeatability for the identified miRNAs in different conditions is needed. Therefore, the goal of our study was to investigate the populations of miRNAs expressed in colorectal cancerous tissue when compared with a healthy adjacent tissue and serum from the same patients, to determine potential new biomarkers for early detection, prediction of patient recovery and future more personalized therapeutic approaches.

## Results

**Fig 1:**
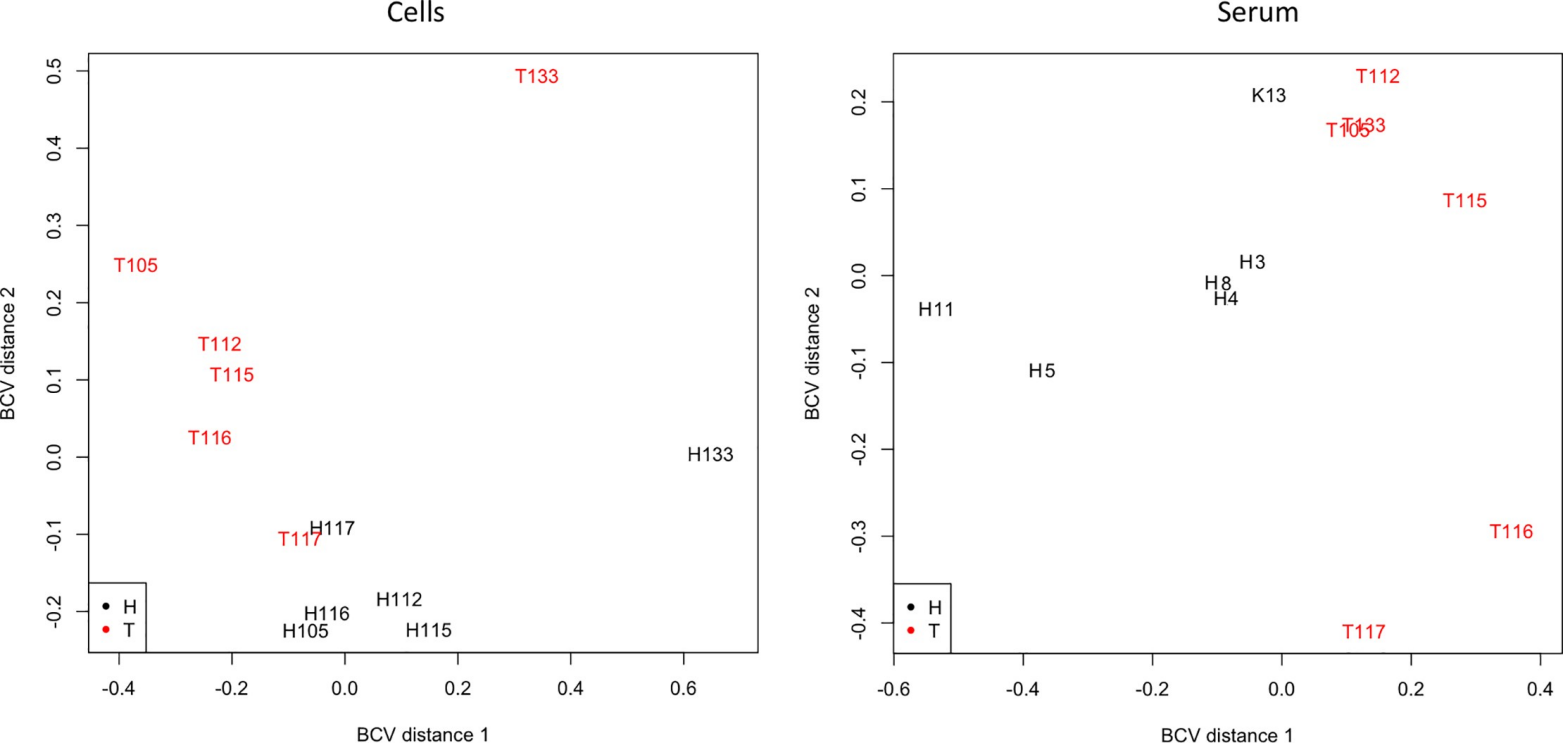
Principal component analysis of the 500 most variable miRNAs in the tissue and serum samples (healthy tissue—H and tumor tissue—T) from patients diagnosed with colorectal cancer.

After sequencing and processing, 12,540,784 adapter cleaned reads/sample with a 64.6% alignment rate to the human genome (hg19) for tissues was obtained in average. In the serum samples, 1,341,762 adapter cleaned reads/sample resulted in a 43.7% alignment rate to the human genome (hg19) in average. Principal component analysis (PCA) from the 500 miRNAs with the most variation in tissue and serum samples indicates a different and very clear pattern of expression between healthy and cancer tissue and serum samples (Fig 1).

Following the initial analysis, the samples with < 3 reads per million (rpm) in more than half of tested samples were removed, which resulted in identification of final 388 different miRNAs expressed in tissue (S1 Table) and 110 miRNAs in the serum samples (S2 Table). Comparison of the expression patterns of miRNAs in tumor and healthy tissue identified 40 differentially expressed miRNAs. Out of these 40 miRNAs, 20 were downregulated, while 20 indicated increased expression (False Discovery rate—FDR<0.05 and Fold Change–FC<0.5 or >2.0; Table 1). For serum samples 8 miRNAs were differentially expressed (4 down- and 4 up-regulated; FDR<0.05 and FC<0.5 or >2.0; Table 2). There was no overlap in the differentially expressed miRNAs between tissue and serum. Only one miRNA regulated in serum was not found as overall expressed in tissue samples (hsa-miR-486-3p), the other seven serum regulated miRNAs were also found in tissue samples, although not differentially regulated.

**Table 1:** MicroRNAs differentially expressed between tumor and healthy adjacent tissue in six patients diagnosed with colorectal cancer.

miRNA^1^	Healthy	Tumor	FC^2^	P Value	FDR^3^
Down-regulated					
hsa-miR-133b	214 ± 309433	13 ± 6	0.06	<0.0001	0.0007
hsa-miR-1-3p	49721 ± 309318	4077 ± 702	0.08	<0.0001	0.0003
hsa-miR-133a-3p	4053 ± 309299	405 ± 151	0.10	<0.0001	0.0003
hsa-miR-363-3p	1440 ± 19226	170 ± 52	0.12	<0.0001	0.0013
hsa-miR-143-3p	2341934 ± 309469	294834 ± 51415	0.13	<0.0001	0.0008
hsa-miR-145-5p	44128 ± 309313	5646 ± 1232	0.13	<0.0001	0.0003
hsa-miR-129-5p	190 ± 19225	25 ± 12	0.13	<0.0001	0.0013
hsa-miR-135a-5p	60 ± 36501	10 ± 4	0.16	0.0012	0.0168
hsa-miR-504-5p	234 ± 309469	42 ± 12	0.18	<0.0001	0.0007
hsa-miR-145-3p	9463 ± 309435	1771 ± 328	0.19	<0.0001	0.0007
hsa-miR-139-3p	60 ± 19225	12 ± 4	0.20	<0.0001	0.0012
hsa-miR-139-5p	949 ± 309434	200 ± 50	0.21	<0.0001	0.0007
hsa-miR-143-5p	10052 ± 19253	2317 ± 547	0.23	0.0003	0.0065
hsa-miR-30c-2-3p	160 ± 19246	43 ± 7	0.27	0.0001	0.0017
hsa-miR-30a-3p	1127 ± 19232	309 ± 76	0.27	<0.0001	0.0015
hsa-miR-195-3p	241 ± 36519	72 ± 10	0.30	0.0009	0.0135
hsa-miR-9-5p	951 ± 19245	293 ± 46	0.31	0.0001	0.0020
hsa-miR-378i	36 ± 36720	11 ± 2	0.32	0.0038	0.0396
hsa-miR-138-5p	40 ± 36749	15 ± 4	0.37	0.0030	0.0325
hsa-miR-378d	900 ± 36720	368 ± 69	0.41	0.0040	0.0406
Up-regulated					
hsa-miR-135b-5p	149 ± 19248	1101 ± 348	7.38	0.0002	0.0048
hsa-miR-592	39 ± 36519	263 ± 107	6.68	0.0004	0.0071
hsa-miR-503-5p	13 ± 36500	64 ± 25	5.03	0.0009	0.0135
hsa-miR-424-5p	57 ± 36501	284 ± 147	4.94	0.0011	0.0159
hsa-miR-514a-3p	12 ± 36766	58 ± 24	4.67	0.0021	0.0261
hsa-miR-584-5p	37 ± 36520	163 ± 54	4.37	0.0004	0.0065
hsa-miR-20a-5p	1869 ± 19237	7150 ± 1639	3.83	0.0001	0.0017
hsa-miR-708-5p	68 ± 36750	257 ± 136	3.76	0.0029	0.0318
hsa-miR-1277-3p	2 ± 36769	10 ± 2	3.65	0.0017	0.0226
hsa-miR-18a-5p	25 ± 19248	87 ± 20	3.54	0.0001	0.0029
hsa-miR-625-3p	115 ± 36769	403 ± 201	3.52	0.0013	0.0179
hsa-miR-224-5p	602 ± 36517	2117 ± 537	3.51	0.0005	0.0081
hsa-miR-21-5p	198267 ± 19264	660219 ± 187444	3.33	0.0004	0.0065
hsa-miR-450b-5p	61 ± 7568	200 ± 67	3.30	0.0044	0.0428
hsa-miR-17-5p	988 ± 19248	2971 ± 572	3.01	0.0003	0.0058
hsa-miR-32-5p	473 ± 36518	1322 ± 180	2.80	0.0004	0.0065
hsa-miR-32-3p	13 ± 36764	35 ± 4	2.62	0.0022	0.0263
hsa-miR-148a-3p	374325 ± 36725	942026 ± 152194	2.52	0.0042	0.0415
hsa-miR-19a-3p	362 ± 36747	879 ± 148	2.43	0.0026	0.0306
hsa-miR-941	357 ± 36749	859 ± 191	2.41	0.0028	0.0314

^1^miRNAs are expressed as reads per million (rpm). miRNA with less than 3 rpm in more than 50% of the samples were removed from analysis.

^2^Fold change in Tumor compared to Healthy tissue

^3^False discovery rate. Only miRNAs with FDR lower than 0.05 were considered as significantly regulated.

**Table 2:** MicroRNAs differentially expressed in serum of tumor and healthy patients diagnosed with colorectal cancer.

miRNA^1^	Healthy	Tumor	FC^2^	PValue	FDR^3^
Down-regulated					
hsa-miR-375	120 ± 22	15 ± 4	0.13	<0.0001	<0.0001
hsa-miR-486-3p	97 ± 11	27 ± 9	0.27	0.0002	0.0056
hsa-miR-486-5p	13664 ± 1286	3995 ± 1097	0.29	<0.0001	0.0010
hsa-miR-1180-3p	20 ± 4	7 ± 1	0.34	0.0035	0.0477
Up-regulated					
hsa-let-7d-5p	87 ± 15	266 ± 79	3.03	0.0010	0.0225
hsa-let-7a-5p	956 ± 146	2569 ± 606	2.69	0.0006	0.0161
hsa-miR-30e-3p	24 ± 2	63 ± 13	2.66	0.0019	0.0342
hsa-let-7f-5p	642 ± 128	1620 ± 415	2.53	0.0034	0.0477

^1^miRNAs are expressed as reads per million (rpm). miRNA with less than 3 rpm in more than 50% of the samples were removed from analysis.

^2^Fold change in Tumor compared to Healthy tissue

^3^False discovery rate. Only miRNAs with FDR lower than 0.05 were considered as significantly regulated.

**Fig 2:**
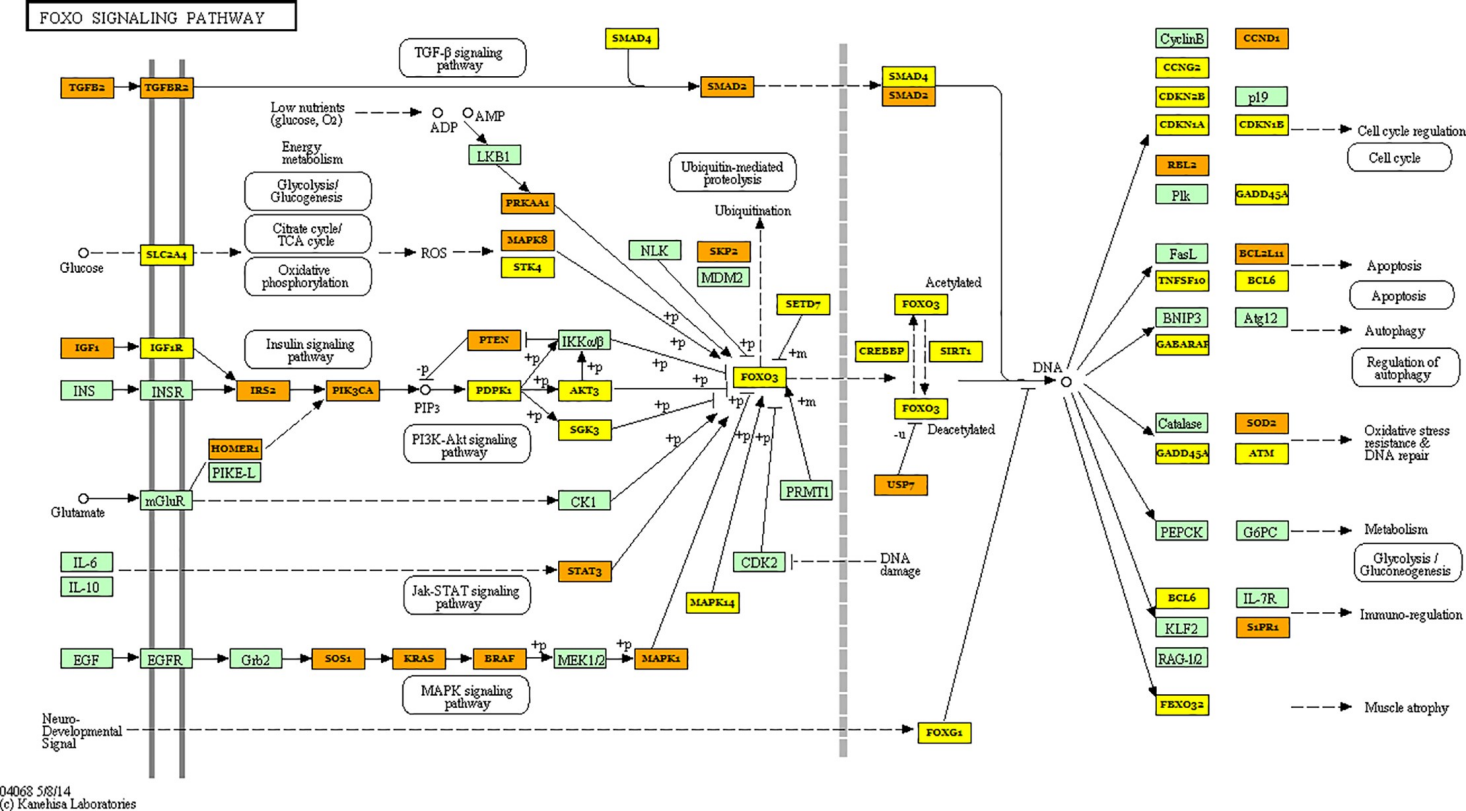
Schematic representation of the FOXO signaling pathway and the target genes of the microRNAs differentially regulated between tumor tissue and healthy tissue from patients diagnosed with colorectal cancer. Yellow box–target gene of one down-regulated miRNA; Orange box–target gene of two or more down-regulated miRNA.

**Fig 3:**
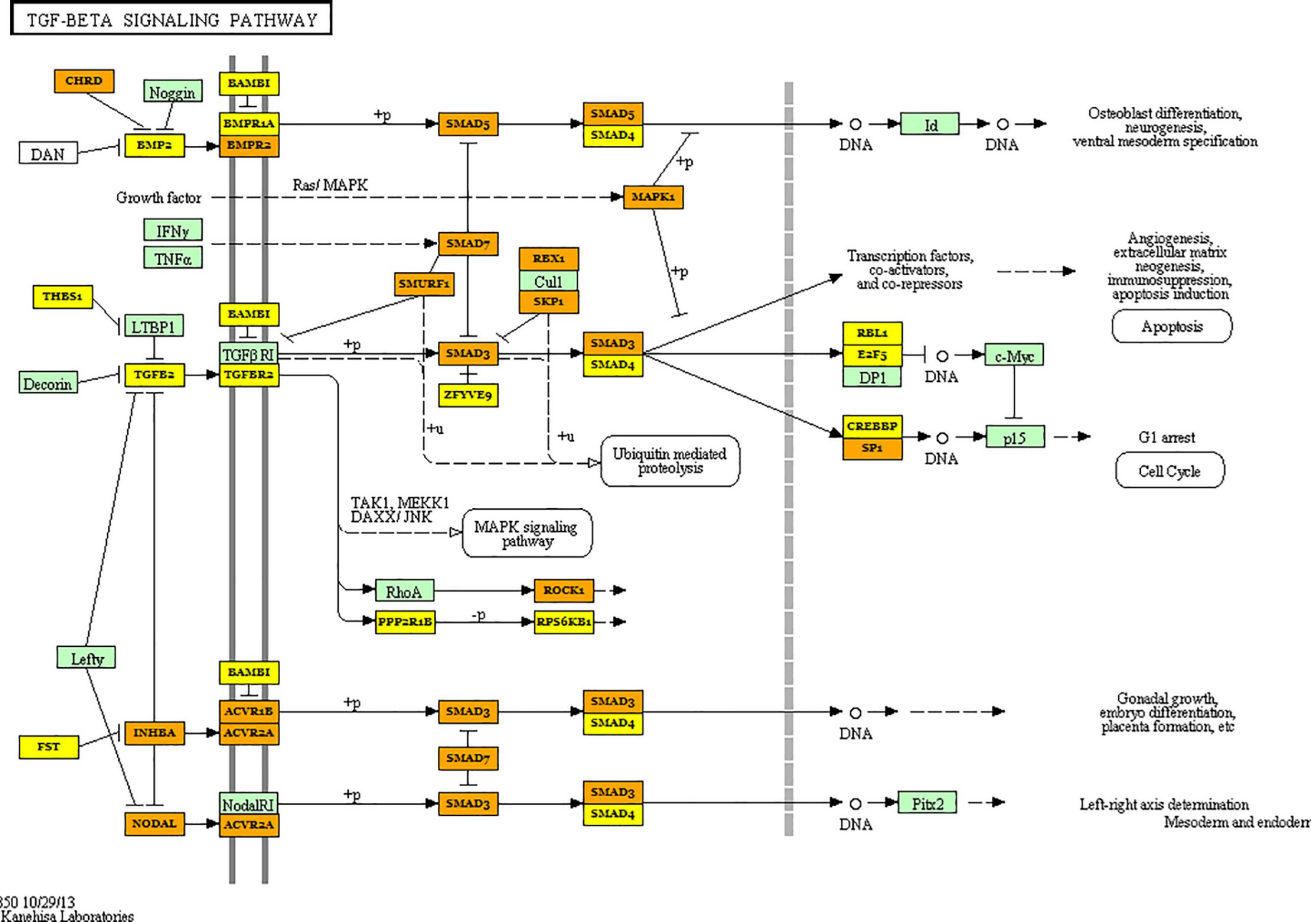
Schematic representation of the TGF-β signaling pathway and the target genes of the microRNAs differentially regulated between tumor tissue and healthy tissue from patients diagnosed with colorectal cancer. Yellow box–target gene of one down-regulated miRNA; Orange box–target gene of two or more down-regulated miRNA.

Pathway and GO term enrichment analysis was performed using the miRNAs differentially regulated in serum (40 miRNAs–see Table 1) and tissue (8 miRNAs–see Table 2) allowed us to identify several known cellular processes regulated by these differentially expressed tissue and serum specific miRNAs. Importantly, the analysis indicated that cancer related pathways are among the top miRNA-regulated pathways in analyzed tissue (Table 3) and serum (Table 4). Additionally, several pathways involving well known oncogenes were significantly targeted by the regulated miRNAs in biopsies samples, as TGF and Foxo signaling pathways (Table 3 and Figs 2 and 3, respectively). GO Terms for biological process and molecular function are presented in S3 and S4 Tables.

**Table 3:** Pathways of target genes from the 40 miRNAs differentially expressed between tumor and healthy tissue of colorectal cancer patients.

KEGG pathway	P value^1^	Genes^2^	miRNAs^3^
Prion diseases	<0.0001	1	2
Morphine addiction	<0.0001	44	10
Mucin type O-Glycan biosynthesis	<0.0001	13	7
ECM-receptor interaction	<0.0001	26	8
Fatty acid biosynthesis	<0.0001	5	1
Signaling pathways regulating pluripotency of stem cells	<0.0001	70	8
TGF-beta signaling pathway	<0.0001	38	8
GABAergic synapse	0.0001	28	8
Axon guidance	0.0001	68	6
Thyroid hormone signaling pathway	0.0006	34	7
Proteoglycans in cancer	0.0007	64	5
Glioma	0.0050	28	7
FoxO signaling pathway	0.0131	56	5
Prolactin signaling pathway	0.01325	42	7
Estrogen signaling pathway	0.02071	24	4
Renal cell carcinoma	0.0211	28	5

^1^Only pathways with P values lower than 0.05 were considered as significant

^2^Number of genes affected in the pathway by the regulated miRNAs

^3^Number of miRNAs differentially expressed that have a target gene in the pathway

**Table 4:** Pathways of target genes from the 8 miRNAs differentially expressed between tumor and healthy serum of colorectal cancer patients.

KEGG pathway	P value^1^	Genes^2^	miRNAs^3^
Prion diseases	<0.0001	1	1
ECM-receptor interaction	<0.0001	10	3
Mucin type O-Glycan biosynthesis	<0.0001	4	3
Signaling pathways regulating pluripotency of stem cells	0.0004	17	3
Thyroid hormone signaling pathway	0.0005	16	4
Biotin metabolism	0.0013	1	1
Amoebiasis	0.0070	11	2
Glycosaminoglycan biosynthesis	0.0279	3	2

^1^Only pathways with P values lower than 0.05 were considered as significant

^2^Number of genes affected in the pathway by the regulated miRNAs

^3^Number of miRNAs differentially expressed that have a target gene in the pathway

## Discussion

In the current study we detected a set of 40 miRNAs differentially regulated in tissue and 8 miRNAs differentially regulated in serum of colorectal cancer patients. There was no overlap in miRNAs regulated in tissue and serum, suggesting that serum regulated miRNAs may be not actively secreted from colorectal tumor cells. However, the differential regulated miRNAs in serum may be leaking passively from damaged cells into circulation [11]. Additionally, this suggests that other cancer driven conditions, i.e. systemic inflammation, oxidative stress, may be driven changes in serum miRNAs to be used as biomarkers.

Some miRNAs are consistently differentially regulated in a myriad of solid cancers (i.e., breast, colon, lung, pancreas, prostate and stomach cancer), with 21 miRNAs identified as regulated in at least three different types of cancer [6]. Interestingly, four of these miRNAs overlapped with miRNAs we currently identified as regulated in colorectal cancer tissue samples, including miR-21, miR-17, miR-20a and miR-32. All these four miRNAs were also identified as differentially expressed in colorectal cancer tissue [6], and miR-21 and miR-17 were identified as regulated in at least five different types of cancer, including breast, lung, prostate, pancreas and stomach [6], suggesting a consistent marker for diagnosis of tumor tissue and involved in carcinogenesis. Additionally, a recent review paper identified several tissue expressed miRNAs associated to poor prognosis in colorectal cancer patients [17]. Our study overlapped with 7 of these identified miRNAs, including miR-21, miR-195, miR-17, miR-20a, miR-145, miR-224 and miR-139. It is interesting that overlapping our study with the previous mentioned studies [6, 17], we can observe that miR-21, miR20a and miR-17 are both predictors of cancer occurrence and poor prognosis in colorectal cancer patients, further indicating their central role in cancer pathogenesis.

Previous studies indicated significant role of miR-21 regulation in colorectal cancer [6, 17]. In present study miR-21-5p was among the highest expressed miRNAs, and more importantly it was significantly upregulated in tumor tissue when comparing with healthy tissue. miR-21 was identified as overexpressed in six different types of cancer [6], and we have previously detected miR-21 as highly abundant and overexpressed in a similar fold change in oral squamous cell carcinoma samples [21]. miR-21 is associated with prognosis of colorectal cancer patients [17], as overexpression of miR-21 shows negative correlation with patients responses to chemotherapy as well as progression-free survival [22]. The central role of miR-21 may be explained by its target genes which include cell growth and proliferation regulating PTEN, a negative regulator of the Pi3k/Akt pathway [23]. Therefore, our study further confirms the central role of miR-21 in cancer development in colorectal patients.

Previous studies have identified 32 miRNAs in serum as regulated in colorectal cancer patients [17]. Comparing to our current study only one miRNA overlapped, miR-375. Others have identified miR-375 as down-regulated in serum of cancer patients, and predictor of cancer recurrence [24], further suggesting its role in diagnosis. In our study miR-375 was ten-fold down-regulated in the serum of cancer patients. A previous paper from our group with oral squamous cell carcinoma patients also identified miR-375 as strongly down-regulated in tissue samples [21]. The hsa-miR-375 is known to target MMP13, which is associated to increased metastatic behavior and cancer aggressiveness [25]. Therefore, it is important to focus more in depth on the role of serum miR-375 in the diagnosis of different types of cancer as well as in the metastatic process, given its target genes and its systemic presence.

We identified miR-143 as strongly down-regulated in serum samples, as others have observed in osteosarcoma, breast cancer and esophageal squamous cell carcinoma [26–28]. miR-143 targets the FOSL2 gene, promoting cell proliferation and metastasis and inhibiting apoptosis [26]. miR-143 constitute a functional cluster along miR-145 [27], which we also identified as down-regulated in our current study, further consolidating both as serum markers for diagnosis. miR-486-5p also was highly expressed and strongly down-regulated in serum of colorectal cancer patients in the current study. miR-485-5p was identified as biomarker of colorectal cancer and malignancy when locally expressed in tumorous tissue [29, 30]. However, serum miR-486-5p was not identified as regulated in a recent review paper on many studies with colorectal cancer patients [17]. One recent study identified both miR-486-3p and -5p as down-regulated in late stage colorectal cancer patients serum but not in early stages patients [31]. This suggests that miR-486 it is not a good marker, as it is not an indicator of early stage cancer, which would constitute a better diagnostic tool for intervention.

miR-148 was strongly up-regulated in serum of colorectal cancer patients in our study. This is controversial, as others have found that miR-148 overexpression inhibited colon cancer cell proliferation and migration [32]. miR-148 expression in tissue samples was down-regulated in a cohort of colorectal cancer patients [33]. More studies are necessary to better understand the role of miR-148, and the effects of cancer type and stage in its regulation to better understand its role in cancer pathogenesis. We also observed that members of the let-7 family were up-regulated in serum of colorectal cancer patients. This is controversial as a previous study has found let-7 to be down-regulated and negatively correlated with metastasis in serum of breast cancer patients [34]. Interestingly, it is suggested that a metastatic gastric cancer line actively secrets members of the let-7 family in the extracellular environment via exosomes to maintain their oncogenesis [35]. Therefore, although let-7 is a tumor suppressor miRNA, its presence in serum may be an indication of increased tumorigenesis and metastatic activity in cancerous tissue, providing a new approach to understand regulation of these biomarkers.

In sum, we detected a set of 40 miRNAs differentially regulated in tissue and 8 miRNAs differentially regulated in serum of colorectal cancer patients. There was no overlap in miRNAs regulated in tissue and serum. Therefore, our study further validates previous miRNAs observed as important in colorectal cancer and other types of cancer and suggests that serum regulated miRNAs may not be the same locally regulated in tissue samples.

## Materials and methods

### Sample and tissue collection

Tissue and serum samples were obtained during surgical procedure from six patients diagnosed with colorectal cancer (4 men and 2 women) with average age of 67.3 years (from 44 to 76 years old). All samples included in the study consisted of tumors in stage G2 (adenocarcinoma tubulare invasivum coli, G2). Recurrences and patients initially treated with radiotherapy were excluded from the study. The details including TNM, Dukes and Astler-Coller classification are presented in Table 5. Additionally, blood samples from six healthy patients were collected for RNA extraction.

**Table 5:** Characteristics of the samples used in the study.

Sample	TNM	Dukes	Astler-Coller
1	pT3, pN2b	C	C2
2	pT3, pN1b	C	C2
3	pT1, pN0	A	B1
4	pT3, pN1b	C	C2
5	pT3, pN0	B	B2
6	pT4a, pN1a	C	C2

Blood samples (n = 12, six colorectal cancer patients and six healthy subjects, never diagnosed with any type of tumor, with the average age of 66.6 years) were collected approximately24hours prior to any surgical intervention, in BD Vacutainer Serum Separation Tubes, incubated 15 minutes in room temperature, centrifuged for serum separation and then stored in -80^o^ C. Additionally, from every colorectal patient two separate tissue specimens were obtained during surgical resection. Core biopsy from the tumor and healthy adjacent tissue within the range of 15–20 cm distal from tumor tissue were collected to allow comparison of tumor site versus non-tumor healthy tissue, in the same cancer patient. Specimens were immediately frozen in liquid nitrogen and then stored in -80 ^o^ C.

This study was carried out in accordance with the recommendations and approval by Institutional Review Board of the University of Medical Sciences in Poznan. All subjects gave written informed consent in accordance with the Declaration of Helsinki.

### RNA extraction and miRNA library preparation

Previously frozen tissues samples (n = 12) were homogenized with Qiazol (Qiagen, Valencia, CA, USA) using zirconium oxide beads (0.5 mm) in the Bullet Blender 24 (Next Advance, Averill Park, NY, USA). Total RNA was extracted from tissue samples using a commercial column purification system (miRNeasy Mini Kit, Qiagen) and on-column DNase treatment (RNase-free DNase Set, Qiagen) following manufacturer's instructions. RNA extraction from serum samples (n = 12) was performed with the miRNEasy Serum/Plasma kit (Qiagen) also following manufacturers instructions.

TruSeq Small RNA Sample Prep Kit (Illumina Inc., San Diego, CA, USA) following the manufacturer's instructions as adjusted by Matkovich, Hu [36] was used to prepare the miRNAs libraries. Briefly, small RNAs from serum and tissue samples total RNA were ligated with 3′ and 5′ adapters, followed by reverse transcription to produce single stranded cDNAs. Adaptor-ligated miRNAs were then amplified by 14 cycles PCR using indexes to allow individual libraries to be processed together in a single flowcell lane during the sequencing step (12 tissue and 12 serum samples). Samples were mixed and a 6% acrylamide gel was used to size-select and purify the amplified libraries.

BioAnalyzer and RNA Nano Lab Chip Kit (Agilent Technologies, Santa Clara, CA, USA) was used to determine the quality and quantity of the libraries. Following the quality check all samples were pooled into one tube and sent for sequencing on a HiSeq 2500 instrument (Illumina Inc.).

### miRNAs libraries analysis and statistical analyses

Alignment and quantification of miRNA libraries was performed using sRNAtoolbox as described before [37]. Statistical analyses of differentially expressed miRNAs was performed using EdgeR [38] on the R software (3.2.2) and miRNAs with a FDR<0.05 and FC>2.0 were considered as up-regulated; and FDR<0.05 and FC<0.50 were considered as down-regulated.

### miRNAs target prediction and enriched pathways and GO Terms

Target genes of the differentially regulated miRNAs were predicted using the mirPath tool (version 3.0) and the microT-CDS v. 5.0 database [39]. Gene ontology (GO) terms (biological processes) and KEGG molecular pathways [40, 41] were also retrieved using the same tool. Pathways and processes regulated with P values lower than 0.05 were considered as significant.

## Supporting information

S1 Table MicroRNAs expressed in serum from tumor and healthy patients diagnosed with colorectal cancer. (DOCX)

S2 Table MicroRNAs expressed in tumor and healthy adjacent tissue in patients diagnosed with colorectal cancer. (DOCX)

S3 Table Gene ontology terms for biological processes, molecular function and cellular compartment of target genes from the 40 miRNAs differentially expressed between tumor and healthy tissue of colorectal cancer patients. (DOCX)

S4 Table Gene ontology terms for biological processes, molecular function and cellular compartment of target genes from the 8 miRNAs differentially expressed between tumor and healthy serum of colorectal cancer patients. (DOCX)
