# Supplementary material for: MicroRNA regulation in colorectal cancer tissue and serum
Source: PLoS One. 2019 Aug 30;14(8):e0222013. doi: 10.1371/journal.pone.0222013 (PMC6716664; doi:10.1371/journal.pone.0222013)
Supplement: S1 Table — (DOCX) [file pone.0222013.s001.docx]

S1 Table – MicroRNAs expressed in serum from tumor and healthy patients diagnosed with colorectal cancer.

| miRNA | Healthy | Tumor | FC | PValue | FDR |
| --- | --- | --- | --- | --- | --- |
| hsa-miR-375 | 120 ± 22 | 15 ± 4 | 0.127 | 1.81E-07 | 1.99E-05 |
| hsa-miR-486-5p | 13664 ± 1286 | 3995 ± 1097 | 0.292 | 1.9E-05 | 1.04E-03 |
| hsa-miR-486-3p | 97 ± 11 | 27 ± 9 | 0.270 | 0.000 | 0.006 |
| hsa-let-7a-5p | 956 ± 146 | 2569 ± 606 | 2.687 | 0.001 | 0.016 |
| hsa-let-7d-5p | 87 ± 15 | 266 ± 79 | 3.031 | 0.001 | 0.022 |
| hsa-miR-30e-3p | 24 ± 2 | 63 ± 13 | 2.657 | 0.002 | 0.034 |
| hsa-let-7f-5p | 642 ± 128 | 1620 ± 415 | 2.526 | 0.003 | 0.048 |
| hsa-miR-1180-3p | 20 ± 4 | 7 ± 1 | 0.337 | 0.003 | 0.048 |
| hsa-miR-199a-5p | 21 ± 7 | 57 ± 9 | 2.662 | 0.005 | 0.054 |
| hsa-miR-26a-5p | 1415 ± 220 | 2909 ± 458 | 2.056 | 0.006 | 0.054 |
| hsa-miR-451a | 6892 ± 1885 | 2824 ± 616 | 0.410 | 0.004 | 0.054 |
| hsa-miR-92a-3p | 3777 ± 281 | 1833 ± 376 | 0.485 | 0.006 | 0.054 |
| hsa-let-7b-5p | 3078 ± 582 | 1569 ± 166 | 0.510 | 0.007 | 0.057 |
| hsa-let-7c-5p | 74 ± 3 | 160 ± 27 | 2.164 | 0.008 | 0.062 |
| hsa-miR-16-5p | 67 ± 14 | 30 ± 4 | 0.445 | 0.009 | 0.068 |
| hsa-miR-25-3p | 976 ± 193 | 495 ± 66 | 0.507 | 0.011 | 0.077 |
| hsa-miR-21-5p | 658 ± 64 | 1288 ± 297 | 1.959 | 0.013 | 0.084 |
| hsa-miR-30c-5p | 58 ± 11 | 121 ± 25 | 2.072 | 0.015 | 0.094 |
| hsa-miR-361-3p | 22 ± 7 | 52 ± 15 | 2.388 | 0.019 | 0.108 |
| hsa-miR-744-5p | 73 ± 18 | 162 ± 15 | 2.199 | 0.020 | 0.108 |
| hsa-let-7i-5p | 844 ± 78 | 1451 ± 227 | 1.718 | 0.025 | 0.108 |
| hsa-miR-126-3p | 721 ± 56 | 1243 ± 185 | 1.724 | 0.023 | 0.108 |
| hsa-miR-222-3p | 243 ± 39 | 454 ± 71 | 1.867 | 0.026 | 0.108 |
| hsa-miR-30a-5p | 46 ± 5 | 24 ± 3 | 0.531 | 0.024 | 0.108 |
| hsa-miR-339-5p | 3 ± 2 | 10 ± 3 | 2.795 | 0.022 | 0.108 |
| hsa-miR-423-3p | 197 ± 23 | 399 ± 129 | 2.020 | 0.026 | 0.108 |
| hsa-miR-98-5p | 26 ± 4 | 58 ± 16 | 2.249 | 0.026 | 0.108 |
| hsa-miR-4446-3p | 7 ± 3 | 21 ± 4 | 2.621 | 0.028 | 0.109 |
| hsa-miR-101-3p | 84 ± 15 | 43 ± 9 | 0.517 | 0.029 | 0.110 |
| hsa-miR-148b-3p | 34 ± 6 | 68 ± 15 | 1.990 | 0.032 | 0.116 |
| hsa-miR-330-3p | 8 ± 2 | 19 ± 2 | 2.195 | 0.034 | 0.119 |
| hsa-miR-192-5p | 229 ± 57 | 118 ± 25 | 0.515 | 0.048 | 0.150 |
| hsa-miR-24-3p | 233 ± 21 | 399 ± 88 | 1.713 | 0.048 | 0.150 |
| hsa-miR-425-5p | 47 ± 7 | 91 ± 24 | 1.927 | 0.045 | 0.150 |
| hsa-miR-7-5p | 23 ± 3 | 45 ± 11 | 1.888 | 0.046 | 0.150 |
| hsa-miR-125b-5p | 36 ± 6 | 18 ± 5 | 0.494 | 0.061 | 0.180 |
| hsa-miR-191-5p | 214 ± 34 | 373 ± 70 | 1.734 | 0.061 | 0.180 |
| hsa-miR-185-5p | 87 ± 14 | 48 ± 14 | 0.555 | 0.064 | 0.185 |
| hsa-let-7g-5p | 261 ± 35 | 437 ± 92 | 1.676 | 0.069 | 0.193 |
| hsa-miR-1301-3p | 11 ± 2 | 21 ± 5 | 1.830 | 0.089 | 0.227 |
| hsa-miR-22-3p | 385 ± 137 | 221 ± 21 | 0.573 | 0.083 | 0.227 |
| hsa-miR-221-3p | 48 ± 17 | 103 ± 45 | 2.131 | 0.088 | 0.227 |
| hsa-miR-532-5p | 103 ± 17 | 63 ± 12 | 0.608 | 0.087 | 0.227 |
| hsa-miR-4433b-5p | 14 ± 5 | 30 ± 12 | 2.119 | 0.100 | 0.244 |
| hsa-miR-92b-3p | 37 ± 7 | 19 ± 7 | 0.501 | 0.099 | 0.244 |
| hsa-miR-16-2-3p | 84 ± 14 | 52 ± 11 | 0.623 | 0.121 | 0.284 |
| hsa-miR-27a-3p | 112 ± 22 | 219 ± 118 | 1.956 | 0.123 | 0.284 |
| hsa-miR-320b | 60 ± 13 | 36 ± 4 | 0.601 | 0.124 | 0.284 |
| hsa-miR-100-5p | 42 ± 7 | 27 ± 4 | 0.643 | 0.140 | 0.315 |
| hsa-miR-103a-3p | 50 ± 13 | 82 ± 18 | 1.635 | 0.144 | 0.316 |
| hsa-miR-194-5p | 30 ± 6 | 18 ± 4 | 0.593 | 0.168 | 0.363 |
| hsa-miR-139-5p | 24 ± 6 | 39 ± 8 | 1.622 | 0.172 | 0.365 |
| hsa-miR-320a | 734 ± 75 | 532 ± 66 | 0.725 | 0.178 | 0.370 |
| hsa-miR-1307-3p | 111 ± 17 | 160 ± 23 | 1.438 | 0.194 | 0.396 |
| hsa-miR-484 | 105 ± 15 | 161 ± 50 | 1.529 | 0.204 | 0.408 |
| hsa-miR-941 | 36 ± 10 | 57 ± 15 | 1.565 | 0.209 | 0.411 |
| hsa-miR-155-5p | 19 ± 4 | 29 ± 5 | 1.518 | 0.216 | 0.417 |
| hsa-miR-99b-5p | 82 ± 7 | 113 ± 15 | 1.380 | 0.223 | 0.424 |
| hsa-let-7e-5p | 25 ± 4 | 37 ± 7 | 1.456 | 0.231 | 0.431 |
| hsa-miR-99a-5p | 363 ± 49 | 251 ± 73 | 0.693 | 0.247 | 0.452 |
| hsa-miR-30e-5p | 152 ± 19 | 107 ± 22 | 0.708 | 0.252 | 0.454 |
| hsa-miR-23a-3p | 19 ± 3 | 28 ± 5 | 1.426 | 0.267 | 0.475 |
| hsa-miR-128-3p | 300 ± 50 | 396 ± 45 | 1.316 | 0.288 | 0.502 |
| hsa-miR-224-5p | 24 ± 15 | 46 ± 16 | 1.839 | 0.292 | 0.502 |
| hsa-miR-182-5p | 31 ± 6 | 46 ± 15 | 1.462 | 0.304 | 0.507 |
| hsa-miR-381-3p | 13 ± 3 | 19 ± 5 | 1.494 | 0.301 | 0.507 |
| hsa-let-7d-3p | 224 ± 20 | 164 ± 48 | 0.732 | 0.318 | 0.514 |
| hsa-miR-151a-5p | 25 ± 11 | 40 ± 10 | 1.559 | 0.313 | 0.514 |
| hsa-miR-151b | 25 ± 11 | 39 ± 10 | 1.544 | 0.325 | 0.518 |
| hsa-miR-378a-3p | 154 ± 24 | 115 ± 28 | 0.751 | 0.336 | 0.528 |
| hsa-miR-93-5p | 20 ± 4 | 28 ± 4 | 1.369 | 0.342 | 0.529 |
| hsa-miR-122-5p | 8647 ± 3201 | 5592 ± 1935 | 0.647 | 0.347 | 0.530 |
| hsa-miR-151a-3p | 1312 ± 251 | 1663 ± 137 | 1.268 | 0.353 | 0.532 |
| hsa-miR-140-3p | 54 ± 11 | 76 ± 20 | 1.412 | 0.362 | 0.538 |
| hsa-miR-10a-5p | 437 ± 31 | 332 ± 92 | 0.760 | 0.376 | 0.552 |
| hsa-miR-146b-5p | 100 ± 11 | 130 ± 30 | 1.305 | 0.394 | 0.571 |
| hsa-miR-584-5p | 29 ± 8 | 40 ± 5 | 1.328 | 0.411 | 0.588 |
| hsa-miR-363-3p | 27 ± 9 | 37 ± 4 | 1.317 | 0.442 | 0.623 |
| hsa-miR-148a-3p | 3037 ± 227 | 3768 ± 1179 | 1.241 | 0.447 | 0.623 |
| hsa-miR-10b-5p | 809 ± 75 | 612 ± 208 | 0.756 | 0.453 | 0.623 |
| hsa-miR-130b-5p | 14 ± 6 | 10 ± 2 | 0.714 | 0.476 | 0.632 |
| hsa-miR-2110 | 23 ± 3 | 17 ± 7 | 0.754 | 0.475 | 0.632 |
| hsa-miR-3615 | 126 ± 30 | 172 ± 104 | 1.362 | 0.477 | 0.632 |
| hsa-miR-197-3p | 21 ± 4 | 16 ± 5 | 0.713 | 0.490 | 0.642 |
| hsa-miR-193a-5p | 32 ± 7 | 24 ± 11 | 0.749 | 0.515 | 0.644 |
| hsa-miR-28-3p | 49 ± 10 | 60 ± 4 | 1.200 | 0.513 | 0.644 |
| hsa-miR-342-5p | 29 ± 6 | 36 ± 7 | 1.258 | 0.513 | 0.644 |
| hsa-miR-629-5p | 22 ± 6 | 29 ± 10 | 1.288 | 0.503 | 0.644 |
| hsa-miR-106b-3p | 29 ± 8 | 36 ± 7 | 1.217 | 0.571 | 0.706 |
| hsa-miR-142-5p | 164 ± 19 | 137 ± 51 | 0.835 | 0.588 | 0.714 |
| hsa-miR-27b-3p | 62 ± 10 | 72 ± 6 | 1.163 | 0.591 | 0.714 |
| hsa-miR-186-5p | 82 ± 12 | 71 ± 15 | 0.861 | 0.617 | 0.723 |
| hsa-miR-199a-3p | 28 ± 1 | 33 ± 10 | 1.214 | 0.605 | 0.723 |
| hsa-miR-26b-5p | 94 ± 19 | 111 ± 23 | 1.170 | 0.618 | 0.723 |
| hsa-miR-127-3p | 128 ± 50 | 162 ± 40 | 1.254 | 0.631 | 0.731 |
| hsa-miR-223-5p | 211 ± 27 | 239 ± 98 | 1.133 | 0.725 | 0.831 |
| hsa-miR-150-5p | 69 ± 6 | 79 ± 40 | 1.139 | 0.740 | 0.839 |
| hsa-miR-143-3p | 931 ± 304 | 829 ± 215 | 0.891 | 0.752 | 0.844 |
| hsa-miR-30d-5p | 1721 ± 73 | 1639 ± 208 | 0.952 | 0.827 | 0.919 |
| hsa-miR-29a-3p | 20 ± 8 | 22 ± 1 | 1.037 | 0.841 | 0.926 |
| hsa-miR-409-3p | 121 ± 53 | 132 ± 43 | 1.084 | 0.865 | 0.933 |
| hsa-miR-423-5p | 2945 ± 158 | 2794 ± 1074 | 0.949 | 0.865 | 0.933 |
| hsa-miR-139-3p | 14 ± 2 | 15 ± 4 | 1.034 | 0.897 | 0.958 |
| hsa-miR-183-5p | 38 ± 9 | 40 ± 16 | 1.034 | 0.912 | 0.965 |
| hsa-miR-125a-5p | 111 ± 12 | 108 ± 32 | 0.972 | 0.945 | 0.974 |
| hsa-miR-146a-5p | 123 ± 29 | 120 ± 24 | 0.977 | 0.957 | 0.974 |
| hsa-miR-181a-5p | 96 ± 19 | 99 ± 19 | 1.025 | 0.935 | 0.974 |
| hsa-miR-328-3p | 103 ± 28 | 100 ± 22 | 0.970 | 0.954 | 0.974 |
| hsa-miR-1246 | 147 ± 34 | 149 ± 38 | 1.011 | 0.967 | 0.975 |
| hsa-miR-671-3p | 12 ± 6 | 12 ± 3 | 0.995 | 1.000 | 1.000 |

^1^miRNAs are expressed as reads per million (rpm). miRNA with less than 3 rpm in less than 50 % of the samples were removed from analysis.

^2^Fold change in Tumor compared to Healthy serum

^3^False discovery rate. Only miRNAs with FDR lower than 0.05 were considered as significantly regulated.
