# Supplementary material for: MicroRNA regulation in colorectal cancer tissue and serum
Source: PLoS One. 2019 Aug 30;14(8):e0222013. doi: 10.1371/journal.pone.0222013 (PMC6716664; doi:10.1371/journal.pone.0222013)
Supplement: S2 Table — (DOCX) [file pone.0222013.s002.docx]

S2 Table – MicroRNAs expressed in tumor and healthy adjacent tissue in patients diagnosed with colorectal cancer.

| miRNA | Healthy | Tumor | FC | PValue | FDR |
| --- | --- | --- | --- | --- | --- |
| hsa-miR-1-3p | 49721 ± 309318 | 4077 ± 702 | 0.082 | 1.28E-06 | 3.22E-04 |
| hsa-miR-133a-3p | 4053 ± 309299 | 405 ± 151 | 0.100 | 2.41E-06 | 3.22E-04 |
| hsa-miR-145-5p | 44128 ± 309313 | 5646 ± 1232 | 0.128 | 2.49E-06 | 3.22E-04 |
| hsa-miR-133b | 214 ± 309433 | 13 ± 6 | 0.061 | 7.78E-06 | 7.03E-04 |
| hsa-miR-139-5p | 949 ± 309434 | 200 ± 50 | 0.210 | 9.18E-06 | 0.001 |
| hsa-miR-145-3p | 9463 ± 309435 | 1771 ± 328 | 0.187 | 1.21E-05 | 0.001 |
| hsa-miR-504-5p | 234 ± 309469 | 42 ± 12 | 0.181 | 1.27E-05 | 0.001 |
| hsa-miR-143-3p | 2341934 ± 309469 | 294834 ± 51415 | 0.126 | 1.58E-05 | 0.001 |
| hsa-miR-139-3p | 60 ± 19225 | 12 ± 4 | 0.196 | 2.83E-05 | 0.001 |
| hsa-miR-129-5p | 190 ± 19225 | 25 ± 12 | 0.132 | 3.40E-05 | 0.001 |
| hsa-miR-363-3p | 1440 ± 19226 | 170 ± 52 | 0.118 | 3.73E-05 | 0.001 |
| hsa-miR-30a-3p | 1127 ± 19232 | 309 ± 76 | 0.274 | 4.54E-05 | 0.001 |
| hsa-miR-20a-5p | 1869 ± 19237 | 7150 ± 1639 | 3.827 | 6.07E-05 | 0.002 |
| hsa-miR-30c-2-3p | 160 ± 19246 | 43 ± 7 | 0.267 | 5.93E-05 | 0.002 |
| hsa-miR-9-5p | 951 ± 19245 | 293 ± 46 | 0.308 | 7.64E-05 | 0.002 |
| hsa-miR-18a-5p | 25 ± 19248 | 87 ± 20 | 3.542 | 1.18E-04 | 0.003 |
| hsa-miR-135b-5p | 149 ± 19248 | 1101 ± 348 | 7.383 | 2.09E-04 | 0.005 |
| hsa-miR-17-5p | 988 ± 19248 | 2971 ± 572 | 3.008 | 2.67E-04 | 0.006 |
| hsa-miR-143-5p | 10052 ± 19253 | 2317 ± 547 | 0.231 | 3.44E-04 | 0.007 |
| hsa-miR-21-5p | 198267 ± 19264 | 660219 ± 187444 | 3.330 | 3.69E-04 | 0.007 |
| hsa-miR-32-5p | 473 ± 36518 | 1322 ± 180 | 2.795 | 3.68E-04 | 0.007 |
| hsa-miR-584-5p | 37 ± 36520 | 163 ± 54 | 4.373 | 3.54E-04 | 0.007 |
| hsa-miR-592 | 39 ± 36519 | 263 ± 107 | 6.682 | 4.19E-04 | 0.007 |
| hsa-miR-224-5p | 602 ± 36517 | 2117 ± 537 | 3.515 | 4.99E-04 | 0.008 |
| hsa-miR-195-3p | 241 ± 36519 | 72 ± 10 | 0.299 | 0.001 | 0.013 |
| hsa-miR-503-5p | 13 ± 36500 | 64 ± 25 | 5.027 | 0.001 | 0.013 |
| hsa-miR-424-5p | 57 ± 36501 | 284 ± 147 | 4.939 | 0.001 | 0.016 |
| hsa-miR-135a-5p | 60 ± 36501 | 10 ± 4 | 0.164 | 0.001 | 0.017 |
| hsa-miR-625-3p | 115 ± 36769 | 403 ± 201 | 3.519 | 0.001 | 0.018 |
| hsa-miR-1277-3p | 2 ± 36769 | 10 ± 2 | 3.649 | 0.002 | 0.023 |
| hsa-miR-514a-3p | 12 ± 36766 | 58 ± 24 | 4.671 | 0.002 | 0.026 |
| hsa-miR-32-3p | 13 ± 36764 | 35 ± 4 | 2.622 | 0.002 | 0.026 |
| hsa-miR-19a-3p | 362 ± 36747 | 879 ± 148 | 2.430 | 0.003 | 0.031 |
| hsa-miR-941 | 357 ± 36749 | 859 ± 191 | 2.406 | 0.003 | 0.031 |
| hsa-miR-708-5p | 68 ± 36750 | 257 ± 136 | 3.761 | 0.003 | 0.032 |
| hsa-miR-138-5p | 40 ± 36749 | 15 ± 4 | 0.374 | 0.003 | 0.032 |
| hsa-miR-378i | 36 ± 36720 | 11 ± 2 | 0.321 | 0.004 | 0.040 |
| hsa-miR-378d | 900 ± 36720 | 368 ± 69 | 0.409 | 0.004 | 0.041 |
| hsa-miR-148a-3p | 374325 ± 36725 | 942026 ± 152194 | 2.517 | 0.004 | 0.042 |
| hsa-miR-450b-5p | 61 ± 7568 | 200 ± 67 | 3.302 | 0.004 | 0.043 |
| hsa-miR-378f | 245 ± 7568 | 98 ± 19 | 0.399 | 0.006 | 0.053 |
| hsa-miR-96-5p | 475 ± 7568 | 1928 ± 790 | 4.056 | 0.006 | 0.053 |
| hsa-miR-18a-3p | 8 ± 7568 | 22 ± 5 | 2.659 | 0.006 | 0.054 |
| hsa-miR-30a-5p | 24999 ± 7566 | 9849 ± 1464 | 0.394 | 0.006 | 0.054 |
| hsa-miR-652-5p | 7 ± 7228 | 23 ± 6 | 3.371 | 0.006 | 0.054 |
| hsa-miR-4791 | 14 ± 7220 | 35 ± 7 | 2.474 | 0.007 | 0.056 |
| hsa-miR-378a-3p | 69632 ± 7220 | 29621 ± 4875 | 0.425 | 0.007 | 0.057 |
| hsa-miR-17-3p | 13 ± 2916 | 36 ± 10 | 2.886 | 0.007 | 0.060 |
| hsa-miR-552-3p | 498 ± 2911 | 1915 ± 680 | 3.846 | 0.008 | 0.061 |
| hsa-miR-106b-5p | 359 ± 2910 | 752 ± 102 | 2.096 | 0.008 | 0.062 |
| hsa-miR-378c | 3042 ± 2912 | 1383 ± 230 | 0.454 | 0.008 | 0.062 |
| hsa-miR-204-5p | 49 ± 2924 | 18 ± 7 | 0.364 | 0.008 | 0.063 |
| hsa-miR-378e | 239 ± 2922 | 104 ± 18 | 0.432 | 0.009 | 0.066 |
| hsa-miR-378g | 249 ± 2923 | 108 ± 18 | 0.432 | 0.009 | 0.066 |
| hsa-miR-99a-5p | 24228 ± 2924 | 7872 ± 2313 | 0.325 | 0.009 | 0.066 |
| hsa-miR-23b-5p | 50 ± 443 | 25 ± 3 | 0.499 | 0.010 | 0.068 |
| hsa-miR-590-3p | 70 ± 443 | 166 ± 37 | 2.355 | 0.010 | 0.068 |
| hsa-miR-27a-5p | 298 ± 441 | 774 ± 261 | 2.601 | 0.012 | 0.079 |
| hsa-miR-374b-3p | 29 ± 441 | 74 ± 18 | 2.548 | 0.013 | 0.083 |
| hsa-miR-193a-5p | 226 ± 548 | 83 ± 29 | 0.367 | 0.013 | 0.087 |
| hsa-miR-335-3p | 552 ± 550 | 1398 ± 486 | 2.532 | 0.014 | 0.087 |
| hsa-miR-4532 | 194 ± 864 | 56 ± 16 | 0.287 | 0.014 | 0.087 |
| hsa-miR-193b-5p | 13 ± 864 | 5 ± 2 | 0.386 | 0.015 | 0.090 |
| hsa-miR-374a-5p | 1528 ± 934 | 3241 ± 556 | 2.121 | 0.015 | 0.090 |
| hsa-miR-552-5p | 99 ± 935 | 348 ± 115 | 3.503 | 0.015 | 0.090 |
| hsa-miR-424-3p | 21 ± 932 | 61 ± 26 | 2.913 | 0.016 | 0.092 |
| hsa-miR-93-5p | 1477 ± 927 | 2849 ± 537 | 1.928 | 0.016 | 0.094 |
| hsa-miR-19b-3p | 916 ± 930 | 1895 ± 346 | 2.069 | 0.017 | 0.094 |
| hsa-miR-4664-3p | 6 ± 935 | 22 ± 6 | 3.448 | 0.017 | 0.094 |
| hsa-let-7c-5p | 3598 ± 931 | 1675 ± 228 | 0.466 | 0.018 | 0.094 |
| hsa-miR-193b-3p | 320 ± 869 | 154 ± 35 | 0.482 | 0.018 | 0.094 |
| hsa-miR-323a-3p | 51 ± 871 | 24 ± 5 | 0.462 | 0.018 | 0.094 |
| hsa-miR-421 | 31 ± 1445 | 73 ± 20 | 2.317 | 0.018 | 0.094 |
| hsa-miR-542-3p | 63 ± 1441 | 185 ± 69 | 2.926 | 0.019 | 0.094 |
| hsa-miR-548o-3p | 41 ± 1446 | 89 ± 16 | 2.165 | 0.017 | 0.094 |
| hsa-miR-660-5p | 566 ± 1446 | 1075 ± 84 | 1.899 | 0.018 | 0.094 |
| hsa-miR-10a-3p | 320 ± 1450 | 742 ± 217 | 2.317 | 0.019 | 0.095 |
| hsa-miR-182-5p | 2822 ± 1451 | 8479 ± 3449 | 3.004 | 0.019 | 0.095 |
| hsa-miR-1247-5p | 21 ± 1430 | 81 ± 25 | 3.771 | 0.021 | 0.098 |
| hsa-miR-125a-5p | 6964 ± 1429 | 3501 ± 362 | 0.503 | 0.023 | 0.098 |
| hsa-miR-149-5p | 138 ± 1297 | 58 ± 16 | 0.421 | 0.020 | 0.098 |
| hsa-miR-203a-3p | 4187 ± 1298 | 13574 ± 5244 | 3.242 | 0.023 | 0.098 |
| hsa-miR-29c-5p | 351 ± 1881 | 182 ± 26 | 0.519 | 0.021 | 0.098 |
| hsa-miR-328-3p | 405 ± 1892 | 213 ± 32 | 0.525 | 0.021 | 0.098 |
| hsa-miR-34a-5p | 803 ± 1893 | 1871 ± 561 | 2.330 | 0.022 | 0.098 |
| hsa-miR-378a-5p | 289 ± 1949 | 139 ± 27 | 0.480 | 0.022 | 0.098 |
| hsa-miR-450a-5p | 61 ± 1945 | 159 ± 46 | 2.580 | 0.023 | 0.098 |
| hsa-miR-497-5p | 414 ± 1943 | 206 ± 29 | 0.498 | 0.020 | 0.098 |
| hsa-miR-500a-5p | 25 ± 1940 | 55 ± 10 | 2.247 | 0.020 | 0.098 |
| hsa-miR-766-3p | 30 ± 1940 | 13 ± 3 | 0.423 | 0.023 | 0.098 |
| hsa-miR-92a-3p | 13460 ± 1939 | 28226 ± 6979 | 2.097 | 0.021 | 0.098 |
| hsa-miR-183-5p | 1162 ± 1611 | 3316 ± 1164 | 2.854 | 0.023 | 0.098 |
| hsa-let-7e-5p | 2290 ± 1614 | 1016 ± 229 | 0.444 | 0.024 | 0.098 |
| hsa-miR-485-5p | 45 ± 1602 | 21 ± 6 | 0.462 | 0.024 | 0.098 |
| hsa-miR-125b-2-3p | 157 ± 1681 | 44 ± 15 | 0.282 | 0.025 | 0.100 |
| hsa-miR-15b-5p | 196 ± 1681 | 350 ± 44 | 1.787 | 0.025 | 0.100 |
| hsa-miR-223-3p | 321 ± 1677 | 812 ± 441 | 2.528 | 0.026 | 0.102 |
| hsa-miR-338-5p | 158 ± 1679 | 71 ± 19 | 0.447 | 0.026 | 0.103 |
| hsa-miR-500b-5p | 8 ± 1681 | 18 ± 2 | 2.348 | 0.027 | 0.107 |
| hsa-let-7e-3p | 33 ± 1681 | 16 ± 3 | 0.499 | 0.030 | 0.115 |
| hsa-miR-23b-3p | 13300 ± 1680 | 6498 ± 905 | 0.489 | 0.030 | 0.115 |
| hsa-miR-29b-3p | 3857 ± 936 | 8141 ± 2133 | 2.111 | 0.030 | 0.115 |
| hsa-miR-452-5p | 265 ± 893 | 481 ± 59 | 1.817 | 0.030 | 0.115 |
| hsa-miR-148b-3p | 7053 ± 896 | 12906 ± 1928 | 1.830 | 0.032 | 0.119 |
| hsa-miR-95-3p | 796 ± 678 | 1863 ± 824 | 2.340 | 0.033 | 0.120 |
| hsa-miR-15a-5p | 187 ± 681 | 350 ± 51 | 1.878 | 0.033 | 0.122 |
| hsa-miR-374a-3p | 907 ± 683 | 2006 ± 622 | 2.211 | 0.034 | 0.123 |
| hsa-miR-433-3p | 26 ± 682 | 13 ± 2 | 0.492 | 0.034 | 0.124 |
| hsa-miR-493-5p | 141 ± 682 | 283 ± 88 | 1.997 | 0.040 | 0.143 |
| hsa-miR-181a-3p | 97 ± 703 | 242 ± 148 | 2.503 | 0.041 | 0.145 |
| hsa-miR-125b-1-3p | 150 ± 705 | 64 ± 16 | 0.424 | 0.042 | 0.147 |
| hsa-miR-155-5p | 1709 ± 706 | 3530 ± 1563 | 2.066 | 0.045 | 0.157 |
| hsa-miR-125b-5p | 5602 ± 703 | 2603 ± 590 | 0.465 | 0.048 | 0.162 |
| hsa-miR-24-1-5p | 157 ± 359 | 80 ± 11 | 0.511 | 0.047 | 0.162 |
| hsa-miR-195-5p | 1058 ± 359 | 576 ± 63 | 0.544 | 0.049 | 0.166 |
| hsa-miR-548av-5p | 17 ± 836 | 32 ± 7 | 1.962 | 0.050 | 0.167 |
| hsa-miR-548k | 17 ± 833 | 32 ± 7 | 1.962 | 0.050 | 0.167 |
| hsa-miR-1255a | 4 ± 833 | 10 ± 2 | 2.290 | 0.058 | 0.185 |
| hsa-miR-144-5p | 97 ± 831 | 250 ± 180 | 2.568 | 0.059 | 0.185 |
| hsa-miR-16-5p | 2523 ± 829 | 4197 ± 532 | 1.664 | 0.059 | 0.185 |
| hsa-miR-1976 | 7 ± 1389 | 19 ± 8 | 2.583 | 0.057 | 0.185 |
| hsa-miR-548e-3p | 21 ± 1387 | 39 ± 9 | 1.871 | 0.059 | 0.185 |
| hsa-miR-651-5p | 105 ± 1386 | 214 ± 49 | 2.037 | 0.057 | 0.185 |
| hsa-miR-7-1-3p | 40 ± 1516 | 73 ± 11 | 1.819 | 0.058 | 0.185 |
| hsa-miR-582-3p | 738 ± 1515 | 362 ± 42 | 0.491 | 0.061 | 0.189 |
| hsa-miR-21-3p | 868 ± 1519 | 1513 ± 253 | 1.743 | 0.065 | 0.200 |
| hsa-miR-708-3p | 56 ± 1524 | 127 ± 65 | 2.289 | 0.065 | 0.200 |
| hsa-miR-218-5p | 2146 ± 1545 | 1076 ± 419 | 0.501 | 0.068 | 0.207 |
| hsa-miR-141-3p | 1289 ± 1549 | 3121 ± 735 | 2.421 | 0.070 | 0.210 |
| hsa-miR-887-3p | 21 ± 2729 | 10 ± 4 | 0.463 | 0.071 | 0.212 |
| hsa-miR-188-5p | 6 ± 2720 | 13 ± 2 | 2.082 | 0.073 | 0.215 |
| hsa-miR-656-3p | 10 ± 2709 | 20 ± 5 | 2.033 | 0.073 | 0.215 |
| hsa-miR-365a-3p | 260 ± 2709 | 137 ± 31 | 0.529 | 0.074 | 0.216 |
| hsa-miR-27a-3p | 8716 ± 2712 | 14705 ± 3002 | 1.687 | 0.076 | 0.220 |
| hsa-let-7a-3p | 367 ± 2666 | 565 ± 47 | 1.543 | 0.079 | 0.225 |
| hsa-miR-4485-3p | 13 ± 2665 | 39 ± 17 | 2.888 | 0.079 | 0.225 |
| hsa-miR-221-5p | 306 ± 2664 | 483 ± 61 | 1.580 | 0.086 | 0.243 |
| hsa-miR-1246 | 373 ± 2665 | 722 ± 202 | 1.937 | 0.087 | 0.244 |
| hsa-miR-28-3p | 11075 ± 12470 | 6546 ± 476 | 0.591 | 0.087 | 0.244 |
| hsa-miR-3613-5p | 223 ± 12482 | 369 ± 60 | 1.656 | 0.088 | 0.244 |
| hsa-miR-1248 | 76 ± 12481 | 157 ± 53 | 2.065 | 0.092 | 0.252 |
| hsa-miR-99b-5p | 7320 ± 12473 | 4215 ± 758 | 0.576 | 0.093 | 0.254 |
| hsa-miR-27b-5p | 138 ± 12494 | 84 ± 9 | 0.611 | 0.097 | 0.263 |
| hsa-miR-106a-5p | 66 ± 14237 | 31 ± 8 | 0.467 | 0.098 | 0.264 |
| hsa-miR-223-5p | 67 ± 14237 | 137 ± 67 | 2.050 | 0.099 | 0.264 |
| hsa-miR-429 | 4221 ± 14238 | 9606 ± 2108 | 2.276 | 0.102 | 0.269 |
| hsa-miR-577 | 31 ± 14261 | 78 ± 36 | 2.548 | 0.102 | 0.269 |
| hsa-miR-200a-3p | 23250 ± 14248 | 54015 ± 11660 | 2.323 | 0.104 | 0.270 |
| hsa-miR-28-5p | 1400 ± 14198 | 858 ± 71 | 0.612 | 0.103 | 0.270 |
| hsa-miR-142-3p | 1611 ± 14183 | 3173 ± 1749 | 1.969 | 0.106 | 0.272 |
| hsa-miR-224-3p | 9 ± 14187 | 17 ± 4 | 1.982 | 0.106 | 0.272 |
| hsa-miR-491-5p | 6 ± 14187 | 11 ± 2 | 1.737 | 0.108 | 0.276 |
| hsa-miR-130a-3p | 404 ± 14186 | 222 ± 51 | 0.550 | 0.110 | 0.278 |
| hsa-miR-148a-5p | 518 ± 14189 | 837 ± 77 | 1.616 | 0.112 | 0.281 |
| hsa-miR-203b-3p | 62 ± 14155 | 151 ± 67 | 2.446 | 0.113 | 0.281 |
| hsa-miR-370-3p | 283 ± 14165 | 170 ± 30 | 0.599 | 0.112 | 0.281 |
| hsa-miR-10a-5p | 126994 ± 14167 | 227009 ± 64991 | 1.788 | 0.117 | 0.290 |
| hsa-miR-337-3p | 31 ± 8812 | 18 ± 3 | 0.608 | 0.123 | 0.300 |
| hsa-miR-99b-3p | 366 ± 8794 | 217 ± 54 | 0.591 | 0.123 | 0.300 |
| hsa-miR-340-5p | 1585 ± 8772 | 2579 ± 871 | 1.627 | 0.125 | 0.303 |
| hsa-let-7i-3p | 34 ± 8784 | 57 ± 13 | 1.670 | 0.127 | 0.303 |
| hsa-miR-10b-5p | 64494 ± 8785 | 38207 ± 5857 | 0.592 | 0.127 | 0.303 |
| hsa-miR-130b-3p | 40 ± 5213 | 66 ± 8 | 1.655 | 0.126 | 0.303 |
| hsa-miR-1247-3p | 26 ± 5212 | 59 ± 22 | 2.229 | 0.130 | 0.304 |
| hsa-miR-324-3p | 90 ± 5201 | 56 ± 8 | 0.631 | 0.129 | 0.304 |
| hsa-miR-374b-5p | 1962 ± 5202 | 3135 ± 683 | 1.598 | 0.130 | 0.304 |
| hsa-miR-153-3p | 18 ± 5215 | 30 ± 5 | 1.675 | 0.136 | 0.315 |
| hsa-miR-29a-3p | 15884 ± 5189 | 24783 ± 4542 | 1.560 | 0.136 | 0.315 |
| hsa-miR-106b-3p | 982 ± 5103 | 1444 ± 122 | 1.471 | 0.141 | 0.319 |
| hsa-miR-532-3p | 44 ± 5095 | 75 ± 15 | 1.679 | 0.140 | 0.319 |
| hsa-miR-6842-3p | 21 ± 5094 | 37 ± 12 | 1.749 | 0.140 | 0.319 |
| hsa-miR-338-3p | 104 ± 5086 | 59 ± 15 | 0.560 | 0.144 | 0.326 |
| hsa-miR-103a-3p | 7910 ± 5087 | 11735 ± 1509 | 1.484 | 0.146 | 0.328 |
| hsa-let-7b-5p | 25090 ± 5103 | 17012 ± 942 | 0.678 | 0.150 | 0.333 |
| hsa-let-7f-2-3p | 62 ± 4711 | 97 ± 8 | 1.562 | 0.151 | 0.333 |
| hsa-miR-200b-3p | 46887 ± 4709 | 99659 ± 27627 | 2.126 | 0.151 | 0.333 |
| hsa-miR-381-3p | 2321 ± 807 | 1619 ± 151 | 0.698 | 0.152 | 0.333 |
| hsa-miR-532-5p | 3662 ± 804 | 5421 ± 578 | 1.481 | 0.153 | 0.333 |
| hsa-miR-1287-5p | 28 ± 761 | 18 ± 5 | 0.634 | 0.169 | 0.364 |
| hsa-miR-362-3p | 7 ± 761 | 12 ± 2 | 1.705 | 0.168 | 0.364 |
| hsa-miR-185-5p | 745 ± 761 | 1173 ± 467 | 1.575 | 0.175 | 0.374 |
| hsa-miR-335-5p | 193 ± 765 | 311 ± 105 | 1.607 | 0.180 | 0.384 |
| hsa-miR-210-3p | 1339 ± 766 | 2157 ± 515 | 1.611 | 0.183 | 0.385 |
| hsa-miR-5571-3p | 16 ± 765 | 28 ± 7 | 1.773 | 0.182 | 0.385 |
| hsa-miR-362-5p | 145 ± 5760 | 222 ± 33 | 1.529 | 0.187 | 0.392 |
| hsa-miR-25-3p | 4652 ± 5760 | 6573 ± 599 | 1.413 | 0.190 | 0.397 |
| hsa-miR-148b-5p | 57 ± 5768 | 88 ± 16 | 1.525 | 0.194 | 0.402 |
| hsa-miR-191-5p | 6990 ± 5763 | 10191 ± 1641 | 1.458 | 0.196 | 0.402 |
| hsa-miR-339-5p | 181 ± 5758 | 265 ± 40 | 1.468 | 0.197 | 0.402 |
| hsa-miR-574-3p | 925 ± 5754 | 659 ± 61 | 0.712 | 0.196 | 0.402 |
| hsa-miR-3913-5p | 10 ± 5757 | 16 ± 2 | 1.600 | 0.200 | 0.407 |
| hsa-miR-98-3p | 33 ± 5757 | 51 ± 11 | 1.542 | 0.202 | 0.407 |
| hsa-miR-345-5p | 183 ± 5948 | 267 ± 28 | 1.458 | 0.205 | 0.412 |
| hsa-miR-24-2-5p | 277 ± 6190 | 416 ± 123 | 1.502 | 0.207 | 0.415 |
| hsa-miR-3615 | 86 ± 6191 | 122 ± 10 | 1.419 | 0.210 | 0.417 |
| hsa-miR-369-3p | 59 ± 6191 | 88 ± 15 | 1.507 | 0.212 | 0.419 |
| hsa-miR-15b-3p | 88 ± 6176 | 128 ± 15 | 1.461 | 0.215 | 0.423 |
| hsa-miR-502-5p | 5 ± 6177 | 9 ± 2 | 1.722 | 0.216 | 0.423 |
| hsa-miR-6130 | 4 ± 6176 | 7 ± 1 | 1.510 | 0.217 | 0.423 |
| hsa-miR-769-3p | 6 ± 6176 | 9 ± 1 | 1.486 | 0.218 | 0.424 |
| hsa-miR-181c-3p | 50 ± 6174 | 75 ± 19 | 1.516 | 0.221 | 0.427 |
| hsa-miR-1260b | 23 ± 6174 | 33 ± 5 | 1.426 | 0.224 | 0.428 |
| hsa-miR-27b-3p | 56478 ± 6174 | 38818 ± 4501 | 0.687 | 0.223 | 0.428 |
| hsa-miR-1307-5p | 220 ± 2736 | 313 ± 56 | 1.428 | 0.225 | 0.429 |
| hsa-miR-582-5p | 94 ± 2729 | 60 ± 10 | 0.642 | 0.233 | 0.442 |
| hsa-miR-196b-5p | 1264 ± 3080 | 1929 ± 636 | 1.526 | 0.236 | 0.443 |
| hsa-miR-454-3p | 133 ± 3072 | 190 ± 36 | 1.431 | 0.236 | 0.443 |
| hsa-miR-425-5p | 1385 ± 4858 | 1925 ± 191 | 1.390 | 0.241 | 0.449 |
| hsa-miR-16-2-3p | 176 ± 4870 | 245 ± 39 | 1.391 | 0.245 | 0.456 |
| hsa-miR-199b-5p | 6310 ± 4846 | 9453 ± 3350 | 1.498 | 0.248 | 0.456 |
| hsa-miR-7-5p | 18433 ± 4873 | 27285 ± 5703 | 1.480 | 0.248 | 0.456 |
| hsa-miR-101-3p | 20396 ± 4677 | 30171 ± 7531 | 1.479 | 0.249 | 0.457 |
| hsa-miR-141-5p | 106 ± 4318 | 204 ± 44 | 1.921 | 0.252 | 0.458 |
| hsa-miR-889-3p | 68 ± 4319 | 101 ± 25 | 1.480 | 0.253 | 0.458 |
| hsa-miR-142-5p | 2612 ± 4507 | 3935 ± 1721 | 1.507 | 0.266 | 0.466 |
| hsa-miR-2110 | 41 ± 4505 | 29 ± 5 | 0.723 | 0.261 | 0.466 |
| hsa-miR-26a-2-3p | 116 ± 4505 | 76 ± 17 | 0.654 | 0.262 | 0.466 |
| hsa-miR-330-5p | 33 ± 4506 | 47 ± 8 | 1.403 | 0.266 | 0.466 |
| hsa-miR-331-3p | 201 ± 4505 | 141 ± 28 | 0.703 | 0.266 | 0.466 |
| hsa-miR-432-5p | 52 ± 4506 | 36 ± 7 | 0.707 | 0.265 | 0.466 |
| hsa-miR-4473 | 16 ± 4506 | 24 ± 9 | 1.548 | 0.259 | 0.466 |
| hsa-miR-652-3p | 620 ± 4497 | 846 ± 116 | 1.364 | 0.266 | 0.466 |
| hsa-miR-200a-5p | 2793 ± 4502 | 5011 ± 1179 | 1.794 | 0.270 | 0.469 |
| hsa-miR-146a-5p | 15822 ± 4516 | 24074 ± 7595 | 1.522 | 0.274 | 0.472 |
| hsa-miR-320a | 3320 ± 4309 | 2427 ± 540 | 0.731 | 0.274 | 0.472 |
| hsa-miR-194-5p | 39053 ± 4321 | 68740 ± 15574 | 1.760 | 0.276 | 0.473 |
| hsa-miR-495-3p | 96 ± 1688 | 68 ± 10 | 0.714 | 0.280 | 0.479 |
| hsa-miR-30e-3p | 3814 ± 1687 | 2811 ± 391 | 0.737 | 0.282 | 0.481 |
| hsa-miR-664a-5p | 30 ± 1686 | 22 ± 5 | 0.730 | 0.285 | 0.483 |
| hsa-miR-500a-3p | 371 ± 1681 | 500 ± 64 | 1.349 | 0.287 | 0.484 |
| hsa-miR-410-3p | 30 ± 1682 | 21 ± 3 | 0.706 | 0.291 | 0.486 |
| hsa-miR-874-3p | 78 ± 2747 | 113 ± 25 | 1.451 | 0.290 | 0.486 |
| hsa-miR-200c-3p | 15750 ± 2748 | 26969 ± 6867 | 1.712 | 0.294 | 0.489 |
| hsa-miR-181a-5p | 5031 ± 2324 | 6836 ± 1537 | 1.359 | 0.311 | 0.516 |
| hsa-miR-5010-3p | 15 ± 2305 | 21 ± 6 | 1.477 | 0.320 | 0.528 |
| hsa-miR-323b-3p | 24 ± 2300 | 16 ± 4 | 0.701 | 0.326 | 0.536 |
| hsa-miR-425-3p | 169 ± 2300 | 222 ± 20 | 1.316 | 0.327 | 0.536 |
| hsa-miR-130b-5p | 70 ± 2298 | 102 ± 25 | 1.462 | 0.329 | 0.537 |
| hsa-miR-1301-3p | 44 ± 2299 | 65 ± 30 | 1.485 | 0.332 | 0.537 |
| hsa-miR-455-5p | 1145 ± 2294 | 882 ± 103 | 0.771 | 0.331 | 0.537 |
| hsa-miR-10b-3p | 64 ± 34699 | 45 ± 8 | 0.706 | 0.338 | 0.544 |
| hsa-miR-29b-2-5p | 24 ± 34699 | 16 ± 5 | 0.688 | 0.339 | 0.544 |
| hsa-miR-1180-3p | 114 ± 34699 | 82 ± 20 | 0.720 | 0.342 | 0.547 |
| hsa-miR-329-3p | 21 ± 34699 | 15 ± 3 | 0.725 | 0.344 | 0.547 |
| hsa-miR-320b | 150 ± 34691 | 113 ± 15 | 0.752 | 0.351 | 0.556 |
| hsa-miR-144-3p | 314 ± 34691 | 441 ± 200 | 1.407 | 0.359 | 0.566 |
| hsa-miR-221-3p | 3823 ± 34690 | 4911 ± 473 | 1.285 | 0.366 | 0.571 |
| hsa-miR-486-5p | 1239 ± 34705 | 923 ± 289 | 0.745 | 0.367 | 0.571 |
| hsa-miR-505-3p | 171 ± 34710 | 220 ± 21 | 1.285 | 0.366 | 0.571 |
| hsa-miR-100-5p | 23622 ± 34711 | 16957 ± 4584 | 0.718 | 0.371 | 0.574 |
| hsa-miR-1271-5p | 27 ± 34739 | 19 ± 6 | 0.697 | 0.373 | 0.574 |
| hsa-miR-501-3p | 123 ± 34703 | 163 ± 20 | 1.329 | 0.372 | 0.574 |
| hsa-let-7f-1-3p | 82 ± 34703 | 106 ± 8 | 1.282 | 0.376 | 0.577 |
| hsa-miR-423-5p | 1481 ± 34703 | 1177 ± 101 | 0.794 | 0.388 | 0.592 |
| hsa-miR-511-5p | 76 ± 35055 | 56 ± 9 | 0.737 | 0.389 | 0.592 |
| hsa-miR-361-5p | 423 ± 35054 | 527 ± 51 | 1.245 | 0.391 | 0.593 |
| hsa-miR-501-5p | 18 ± 35056 | 26 ± 6 | 1.412 | 0.393 | 0.593 |
| hsa-miR-181b-5p | 1071 ± 35056 | 1373 ± 293 | 1.283 | 0.396 | 0.595 |
| hsa-miR-192-5p | 324906 ± 35057 | 507839 ± 143933 | 1.563 | 0.400 | 0.599 |
| hsa-miR-134-5p | 230 ± 6897 | 176 ± 34 | 0.766 | 0.404 | 0.603 |
| hsa-miR-1275 | 20 ± 6897 | 29 ± 6 | 1.405 | 0.423 | 0.626 |
| hsa-miR-3158-3p | 39 ± 6893 | 52 ± 17 | 1.328 | 0.421 | 0.626 |
| hsa-miR-128-3p | 1786 ± 6892 | 2242 ± 468 | 1.255 | 0.425 | 0.626 |
| hsa-miR-382-3p | 49 ± 6900 | 63 ± 16 | 1.309 | 0.426 | 0.626 |
| hsa-miR-197-3p | 588 ± 7011 | 473 ± 35 | 0.805 | 0.432 | 0.631 |
| hsa-miR-214-5p | 272 ± 7003 | 213 ± 48 | 0.784 | 0.431 | 0.631 |
| hsa-miR-382-5p | 117 ± 7005 | 90 ± 17 | 0.765 | 0.435 | 0.632 |
| hsa-miR-299-3p | 14 ± 7006 | 10 ± 3 | 0.747 | 0.437 | 0.632 |
| hsa-miR-30c-1-3p | 32 ± 7006 | 26 ± 3 | 0.795 | 0.439 | 0.632 |
| hsa-miR-146b-5p | 14000 ± 6985 | 18448 ± 7810 | 1.318 | 0.443 | 0.633 |
| hsa-miR-340-3p | 52 ± 6945 | 77 ± 47 | 1.483 | 0.444 | 0.633 |
| hsa-miR-411-5p | 169 ± 6933 | 218 ± 62 | 1.292 | 0.441 | 0.633 |
| hsa-let-7i-5p | 71651 ± 6933 | 94032 ± 36560 | 1.312 | 0.446 | 0.634 |
| hsa-miR-181d-5p | 306 ± 1788 | 390 ± 93 | 1.275 | 0.453 | 0.641 |
| hsa-miR-4284 | 13 ± 1785 | 18 ± 5 | 1.363 | 0.458 | 0.646 |
| hsa-miR-185-3p | 31 ± 1785 | 40 ± 16 | 1.312 | 0.462 | 0.649 |
| hsa-miR-107 | 918 ± 6855 | 1102 ± 60 | 1.200 | 0.475 | 0.661 |
| hsa-miR-196a-5p | 661 ± 6861 | 926 ± 224 | 1.401 | 0.475 | 0.661 |
| hsa-miR-4516 | 81 ± 6865 | 121 ± 55 | 1.485 | 0.474 | 0.661 |
| hsa-miR-29c-3p | 803 ± 6865 | 649 ± 72 | 0.809 | 0.480 | 0.665 |
| hsa-miR-181a-2-3p | 164 ± 19549 | 201 ± 27 | 1.222 | 0.482 | 0.666 |
| hsa-miR-146b-3p | 160 ± 19522 | 203 ± 79 | 1.272 | 0.502 | 0.680 |
| hsa-miR-151a-3p | 18794 ± 19474 | 22738 ± 2706 | 1.210 | 0.503 | 0.680 |
| hsa-miR-222-3p | 1951 ± 19510 | 2357 ± 374 | 1.209 | 0.496 | 0.680 |
| hsa-miR-25-5p | 11 ± 19521 | 14 ± 4 | 1.322 | 0.501 | 0.680 |
| hsa-miR-487b-3p | 39 ± 19494 | 31 ± 6 | 0.791 | 0.497 | 0.680 |
| hsa-miR-671-3p | 32 ± 19494 | 40 ± 8 | 1.239 | 0.503 | 0.680 |
| hsa-miR-127-3p | 5820 ± 19491 | 4800 ± 670 | 0.825 | 0.507 | 0.683 |
| hsa-miR-150-5p | 1117 ± 19518 | 881 ± 321 | 0.789 | 0.516 | 0.693 |
| hsa-miR-361-3p | 2451 ± 19525 | 2926 ± 580 | 1.194 | 0.520 | 0.695 |
| hsa-miR-499a-5p | 74 ± 19539 | 93 ± 19 | 1.257 | 0.521 | 0.695 |
| hsa-miR-200b-5p | 293 ± 19523 | 415 ± 109 | 1.415 | 0.525 | 0.698 |
| hsa-miR-1307-3p | 969 ± 19983 | 1149 ± 108 | 1.186 | 0.534 | 0.705 |
| hsa-miR-5701 | 14 ± 20248 | 19 ± 3 | 1.280 | 0.533 | 0.705 |
| hsa-let-7f-5p | 69893 ± 20245 | 84197 ± 20794 | 1.205 | 0.555 | 0.730 |
| hsa-miR-3182 | 42 ± 19620 | 32 ± 11 | 0.761 | 0.565 | 0.741 |
| hsa-miR-494-3p | 67 ± 19617 | 81 ± 12 | 1.203 | 0.577 | 0.754 |
| hsa-miR-342-5p | 41 ± 19616 | 32 ± 15 | 0.803 | 0.580 | 0.755 |
| hsa-miR-26a-5p | 184881 ± 19572 | 157312 ± 10409 | 0.851 | 0.592 | 0.765 |
| hsa-miR-423-3p | 4895 ± 7831 | 5699 ± 587 | 1.164 | 0.591 | 0.765 |
| hsa-miR-23a-3p | 10721 ± 7868 | 12394 ± 1684 | 1.156 | 0.595 | 0.767 |
| hsa-let-7d-5p | 1870 ± 7909 | 1615 ± 231 | 0.863 | 0.600 | 0.769 |
| hsa-miR-190a-5p | 122 ± 7924 | 149 ± 36 | 1.228 | 0.604 | 0.769 |
| hsa-miR-30b-5p | 4668 ± 7924 | 5408 ± 728 | 1.159 | 0.603 | 0.769 |
| hsa-miR-493-3p | 123 ± 7950 | 145 ± 23 | 1.177 | 0.602 | 0.769 |
| hsa-miR-409-3p | 458 ± 7951 | 390 ± 50 | 0.852 | 0.607 | 0.769 |
| hsa-miR-379-5p | 742 ± 8566 | 640 ± 156 | 0.863 | 0.617 | 0.780 |
| hsa-miR-30b-3p | 16 ± 8573 | 13 ± 3 | 0.818 | 0.623 | 0.783 |
| hsa-miR-331-5p | 28 ± 8571 | 34 ± 4 | 1.181 | 0.623 | 0.783 |
| hsa-miR-98-5p | 2813 ± 8567 | 3246 ± 825 | 1.154 | 0.639 | 0.800 |
| hsa-miR-215-5p | 51964 ± 8890 | 39799 ± 17144 | 0.766 | 0.642 | 0.800 |
| hsa-let-7g-5p | 57588 ± 7447 | 65854 ± 14093 | 1.144 | 0.649 | 0.805 |
| hsa-miR-194-3p | 410 ± 5758 | 523 ± 135 | 1.276 | 0.648 | 0.805 |
| hsa-miR-30d-5p | 35219 ± 5739 | 39855 ± 4365 | 1.132 | 0.656 | 0.811 |
| hsa-miR-147b | 514 ± 4992 | 426 ± 100 | 0.830 | 0.670 | 0.823 |
| hsa-miR-744-5p | 183 ± 4996 | 159 ± 28 | 0.868 | 0.670 | 0.823 |
| hsa-miR-30c-5p | 6563 ± 4997 | 5727 ± 1367 | 0.872 | 0.677 | 0.828 |
| hsa-miR-1296-5p | 97 ± 4995 | 85 ± 13 | 0.871 | 0.680 | 0.830 |
| hsa-miR-296-5p | 20 ± 4993 | 18 ± 5 | 0.849 | 0.690 | 0.839 |
| hsa-miR-320c | 44 ± 4993 | 39 ± 7 | 0.874 | 0.692 | 0.839 |
| hsa-miR-4443 | 59 ± 4993 | 50 ± 15 | 0.845 | 0.695 | 0.839 |
| hsa-miR-4492 | 91 ± 4993 | 124 ± 71 | 1.348 | 0.696 | 0.839 |
| hsa-miR-140-5p | 542 ± 4992 | 603 ± 163 | 1.114 | 0.713 | 0.844 |
| hsa-miR-369-5p | 12 ± 5005 | 11 ± 2 | 0.885 | 0.711 | 0.844 |
| hsa-miR-375 | 34771 ± 5004 | 42718 ± 12815 | 1.229 | 0.708 | 0.844 |
| hsa-miR-3934-5p | 12 ± 3398 | 14 ± 1 | 1.173 | 0.714 | 0.844 |
| hsa-miR-502-3p | 196 ± 3398 | 220 ± 33 | 1.120 | 0.711 | 0.844 |
| hsa-miR-92b-3p | 505 ± 3392 | 456 ± 41 | 0.902 | 0.713 | 0.844 |
| hsa-let-7a-5p | 32799 ± 3396 | 29476 ± 4257 | 0.899 | 0.723 | 0.853 |
| hsa-miR-655-3p | 17 ± 1106 | 15 ± 3 | 0.890 | 0.729 | 0.858 |
| hsa-miR-126-5p | 772 ± 1105 | 851 ± 170 | 1.102 | 0.736 | 0.863 |
| hsa-miR-199a-5p | 7367 ± 1108 | 6598 ± 2043 | 0.896 | 0.743 | 0.868 |
| hsa-miR-324-5p | 104 ± 972 | 94 ± 12 | 0.910 | 0.745 | 0.868 |
| hsa-miR-758-3p | 17 ± 972 | 15 ± 4 | 0.911 | 0.754 | 0.876 |
| hsa-miR-212-5p | 28 ± 1281 | 25 ± 7 | 0.902 | 0.759 | 0.876 |
| hsa-miR-484 | 567 ± 1676 | 522 ± 50 | 0.922 | 0.761 | 0.876 |
| hsa-miR-629-5p | 279 ± 1681 | 257 ± 32 | 0.920 | 0.759 | 0.876 |
| hsa-miR-654-5p | 20 ± 1682 | 18 ± 4 | 0.874 | 0.771 | 0.885 |
| hsa-miR-1291 | 47 ± 1675 | 41 ± 10 | 0.881 | 0.787 | 0.885 |
| hsa-miR-132-5p | 115 ± 1675 | 105 ± 27 | 0.915 | 0.782 | 0.885 |
| hsa-miR-136-5p | 241 ± 2622 | 221 ± 38 | 0.918 | 0.787 | 0.885 |
| hsa-miR-30e-5p | 8730 ± 2625 | 9462 ± 611 | 1.084 | 0.774 | 0.885 |
| hsa-miR-34c-5p | 104 ± 2572 | 94 ± 17 | 0.911 | 0.778 | 0.885 |
| hsa-miR-589-5p | 53 ± 2571 | 58 ± 6 | 1.085 | 0.783 | 0.885 |
| hsa-miR-671-5p | 72 ± 2571 | 80 ± 12 | 1.108 | 0.776 | 0.885 |
| hsa-miR-186-5p | 4047 ± 2567 | 3776 ± 224 | 0.933 | 0.797 | 0.894 |
| hsa-miR-942-5p | 19 ± 2575 | 21 ± 3 | 1.116 | 0.809 | 0.905 |
| hsa-let-7b-3p | 127 ± 2572 | 134 ± 11 | 1.053 | 0.849 | 0.905 |
| hsa-miR-127-5p | 185 ± 2567 | 172 ± 35 | 0.934 | 0.834 | 0.905 |
| hsa-miR-136-3p | 178 ± 2569 | 191 ± 35 | 1.077 | 0.825 | 0.905 |
| hsa-miR-152-3p | 4406 ± 2569 | 4129 ± 1295 | 0.937 | 0.843 | 0.905 |
| hsa-miR-181c-5p | 158 ± 2570 | 171 ± 34 | 1.077 | 0.835 | 0.905 |
| hsa-miR-22-3p | 9561 ± 2571 | 9010 ± 1399 | 0.942 | 0.849 | 0.905 |
| hsa-miR-24-3p | 13128 ± 2467 | 13879 ± 1306 | 1.057 | 0.838 | 0.905 |
| hsa-miR-26b-3p | 25 ± 2207 | 26 ± 1 | 1.067 | 0.848 | 0.905 |
| hsa-miR-339-3p | 140 ± 2207 | 132 ± 17 | 0.943 | 0.843 | 0.905 |
| hsa-miR-342-3p | 864 ± 2207 | 925 ± 360 | 1.071 | 0.838 | 0.905 |
| hsa-miR-409-5p | 41 ± 3697 | 44 ± 9 | 1.075 | 0.831 | 0.905 |
| hsa-miR-451a | 22378 ± 3696 | 20712 ± 9118 | 0.926 | 0.844 | 0.905 |
| hsa-miR-485-3p | 9 ± 3745 | 8 ± 1 | 0.888 | 0.817 | 0.905 |
| hsa-miR-543 | 52 ± 3740 | 57 ± 8 | 1.077 | 0.825 | 0.905 |
| hsa-miR-654-3p | 178 ± 3741 | 190 ± 43 | 1.066 | 0.841 | 0.905 |
| hsa-miR-664a-3p | 89 ± 3742 | 96 ± 20 | 1.073 | 0.833 | 0.905 |
| hsa-miR-769-5p | 496 ± 4497 | 520 ± 26 | 1.049 | 0.843 | 0.905 |
| hsa-miR-215-3p | 129 ± 4501 | 116 ± 53 | 0.897 | 0.853 | 0.906 |
| hsa-miR-4488 | 321 ± 4499 | 360 ± 178 | 1.120 | 0.868 | 0.920 |
| hsa-let-7d-3p | 783 ± 4501 | 814 ± 83 | 1.039 | 0.881 | 0.931 |
| hsa-miR-154-5p | 12 ± 4492 | 11 ± 2 | 0.976 | 0.893 | 0.933 |
| hsa-miR-22-5p | 163 ± 4476 | 171 ± 39 | 1.047 | 0.887 | 0.933 |
| hsa-miR-326 | 126 ± 4477 | 132 ± 37 | 1.046 | 0.892 | 0.933 |
| hsa-miR-3687 | 88 ± 4573 | 96 ± 23 | 1.085 | 0.893 | 0.933 |
| hsa-miR-376c-3p | 40 ± 4675 | 38 ± 7 | 0.960 | 0.895 | 0.933 |
| hsa-miR-214-3p | 699 ± 4782 | 727 ± 168 | 1.040 | 0.906 | 0.942 |
| hsa-miR-330-3p | 51 ± 4905 | 53 ± 12 | 1.036 | 0.912 | 0.945 |
| hsa-miR-455-3p | 71 ± 5026 | 69 ± 15 | 0.964 | 0.914 | 0.945 |
| hsa-miR-26b-5p | 31161 ± 5154 | 32016 ± 2387 | 1.027 | 0.925 | 0.955 |
| hsa-miR-30d-3p | 85 ± 4249 | 82 ± 14 | 0.971 | 0.930 | 0.957 |
| hsa-miR-126-3p | 24361 ± 4385 | 24743 ± 5295 | 1.016 | 0.958 | 0.978 |
| hsa-miR-140-3p | 5606 ± 3759 | 5512 ± 1328 | 0.983 | 0.954 | 0.978 |
| hsa-miR-301a-5p | 9 ± 3960 | 10 ± 1 | 1.015 | 0.961 | 0.978 |
| hsa-miR-576-5p | 44 ± 4159 | 45 ± 4 | 1.022 | 0.958 | 0.978 |
| hsa-miR-199a-3p | 30484 ± 4390 | 30927 ± 9310 | 1.015 | 0.964 | 0.980 |
| hsa-miR-598-3p | 95 ± 602 | 93 ± 16 | 0.985 | 0.968 | 0.981 |
| hsa-miR-132-3p | 269 ± 640 | 271 ± 48 | 1.010 | 0.975 | 0.985 |
| hsa-miR-7706 | 63 ± 693 | 63 ± 8 | 1.012 | 0.986 | 0.994 |
| hsa-miR-151a-5p | 2526 ± 730 | 2524 ± 371 | 0.999 | 0.998 | 1.000 |
| hsa-miR-151b | 2524 ± 711 | 2519 ± 369 | 0.998 | 0.995 | 1.000 |
| hsa-miR-93-3p | 31 ± 9 | 31 ± 4 | 1.016 | 1.000 | 1.000 |

^1^miRNAs are expressed as reads per million (rpm). miRNA with less than 3 rpm in less than 50 % of the samples were removed from analysis.

^2^Fold change in Tumor compared to Healthy tissue

^3^False discovery rate. Only miRNAs with FDR lower than 0.05 were considered as significantly regulated.
