# Supplementary material for: MicroRNA regulation in colorectal cancer tissue and serum
Source: PLoS One. 2019 Aug 30;14(8):e0222013. doi: 10.1371/journal.pone.0222013 (PMC6716664; doi:10.1371/journal.pone.0222013)
Supplement: S3 Table — (DOCX) [file pone.0222013.s003.docx]

S3 Table – Gene ontology terms for biological processes, molecular function and cellular compartment of target genes from the 40 miRNAs differentially expressed between tumor and healthy tissue of colorectal cancer patients.

| GO Term Category | P-value | Genes | miRNAs |
| --- | --- | --- | --- |
| organelle | <0.0001 | 4592 | 35 |
| ion binding | <0.0001 | 2809 | 33 |
| cellular nitrogen compound metabolic process | <0.0001 | 2194 | 32 |
| cellular protein modification process | <0.0001 | 1101 | 31 |
| biosynthetic process | <0.0001 | 1800 | 29 |
| gene expression | <0.0001 | 305 | 23 |
| nucleic acid binding transcription factor activity | <0.0001 | 487 | 22 |
| neurotrophin TRK receptor signaling pathway | <0.0001 | 155 | 21 |
| enzyme binding | <0.0001 | 548 | 20 |
| protein complex | <0.0001 | 1392 | 19 |
| Fc-epsilon receptor signaling pathway | <0.0001 | 94 | 18 |
| protein binding transcription factor activity | <0.0001 | 232 | 17 |
| epidermal growth factor receptor signaling pathway | <0.0001 | 110 | 17 |
| cytosol | <0.0001 | 1010 | 16 |
| cytoskeletal protein binding | <0.0001 | 327 | 15 |
| blood coagulation | <0.0001 | 205 | 15 |
| symbiosis, encompassing mutualism through parasitism | <0.0001 | 215 | 14 |
| nucleoplasm | <0.0001 | 414 | 13 |
| viral process | <0.0001 | 183 | 13 |
| fibroblast growth factor receptor signaling pathway | <0.0001 | 90 | 12 |
| catabolic process | <0.0001 | 606 | 11 |
| synaptic transmission | <0.0001 | 169 | 11 |
| cellular lipid metabolic process | <0.0001 | 69 | 11 |
| transcription, DNA-templated | <0.0001 | 709 | 10 |
| small molecule metabolic process | <0.0001 | 691 | 10 |
| cellular component assembly | <0.0001 | 435 | 10 |
| enzyme regulator activity | <0.0001 | 298 | 10 |
| macromolecular complex assembly | <0.0001 | 286 | 10 |
| mitotic cell cycle | <0.0001 | 125 | 9 |
| transcription initiation from RNA polymerase II promoter | <0.0001 | 75 | 9 |
| cell death | <0.0001 | 302 | 8 |
| cell-cell signaling | <0.0001 | 196 | 8 |
| phosphatidylinositol-mediated signaling | <0.0001 | 53 | 8 |
| response to stress | <0.0001 | 567 | 7 |
| RNA binding | 0.0001 | 407 | 7 |
| nucleobase-containing compound catabolic process | <0.0001 | 237 | 6 |
| protein complex assembly | <0.0001 | 171 | 6 |
| nervous system development | <0.0001 | 132 | 6 |
| cellular protein metabolic process | 0.0007 | 115 | 6 |
| transmembrane transporter activity | 0.0236 | 205 | 5 |
| membrane organization | 0.0008 | 143 | 4 |
| cell motility | 0.0119 | 126 | 4 |
| platelet activation | <0.0001 | 62 | 4 |
| Fc-gamma receptor signaling pathway involved in phagocytosis | 0.0125 | 25 | 4 |
| axon guidance | 0.0072 | 108 | 3 |
| post-translational protein modification | 0.0095 | 41 | 3 |
