# Supplementary material for: MicroRNA regulation in colorectal cancer tissue and serum
Source: PLoS One. 2019 Aug 30;14(8):e0222013. doi: 10.1371/journal.pone.0222013 (PMC6716664; doi:10.1371/journal.pone.0222013)
Supplement: S4 Table — (DOCX) [file pone.0222013.s004.docx]

S4 Table – Gene ontology terms for biological processes, molecular function and cellular compartment of target genes from the 8 miRNAs differentially expressed between tumor and healthy serum of colorectal cancer patients.

| GO Term Category | P-value | Genes | miRNAs |
| --- | --- | --- | --- |
| organelle | 0.000 | 980 | 7 |
| cellular protein modification process | 0.000 | 297 | 7 |
| ion binding | 0.000 | 583 | 6 |
| cellular nitrogen compound metabolic process | 0.000 | 475 | 6 |
| biosynthetic process | 0.000 | 414 | 6 |
| neurotrophin TRK receptor signaling pathway | 0.000 | 42 | 6 |
| Fc-epsilon receptor signaling pathway | 0.000 | 29 | 5 |
| cellular component assembly | 0.000 | 96 | 4 |
| protein binding transcription factor activity | 0.000 | 60 | 4 |
| gene expression | 0.000 | 59 | 4 |
| epidermal growth factor receptor signaling pathway | 0.000 | 34 | 4 |
| phosphatidylinositol-mediated signaling | 0.000 | 18 | 4 |
| macromolecular complex assembly | 0.009 | 43 | 3 |
| miRNA binding | 0.002 | 7 | 3 |
| cell junction assembly | 0.027 | 7 | 3 |
| catabolic process | 0.007 | 71 | 2 |
| nucleic acid binding transcription factor activity | 0.000 | 64 | 2 |
| post-translational protein modification | 0.008 | 12 | 2 |
| cellular response to amino acid stimulus | 0.044 | 11 | 2 |
| extracellular matrix disassembly | 0.014 | 9 | 2 |
| glycosaminoglycan metabolic process | 0.040 | 9 | 2 |
| protein complex | 0.000 | 128 | 1 |
| nucleoplasm | 0.034 | 26 | 1 |
| synaptic transmission | 0.021 | 25 | 1 |
| transcription initiation from RNA polymerase II promoter | 0.033 | 11 | 1 |
| fibroblast growth factor receptor signaling pathway | 0.010 | 9 | 1 |
